# Synthetic and Marine-Derived Porous Scaffolds for Bone Tissue Engineering

**DOI:** 10.3390/ma11091702

**Published:** 2018-09-13

**Authors:** Ana S. Neto, José M. F. Ferreira

**Affiliations:** Department of Materials and Ceramic Engineering, CICECO, University of Aveiro, 3810-193 Aveiro, Portugal; sofia.neto@ua.pt

**Keywords:** bone tissue engineering, biomaterials, bone scaffolds, additive manufacturing techniques/robocasting, marine-derived biomaterials

## Abstract

Bone is a vascularized and connective tissue. The cortical bone is the main part responsible for the support and protection of the remaining systems and organs of the body. The trabecular spongy bone serves as the storage of ions and bone marrow. As a dynamic tissue, bone is in a constant remodelling process to adapt to the mechanical demands and to repair small lesions that may occur. Nevertheless, due to the increased incidence of bone disorders, the need for bone grafts has been growing over the past decades and the development of an ideal bone graft with optimal properties remains a clinical challenge. This review addresses the bone properties (morphology, composition, and their repair and regeneration capacity) and puts the focus on the potential strategies for developing bone repair and regeneration materials. It describes the requirements for designing a suitable scaffold material, types of materials (polymers, ceramics, and composites), and techniques to obtain the porous structures (additive manufacturing techniques like robocasting or derived from marine skeletons) for bone tissue engineering applications. Overall, the main objective of this review is to gather the knowledge on the materials and methods used for the production of scaffolds for bone tissue engineering and to highlight the potential of natural porous structures such as marine skeletons as promising alternative bone graft substitute materials without any further mineralogical changes, or after partial or total transformation into calcium phosphate.

## 1. Introduction

Bone is a vascularized connective tissue responsible for the support and protection of the remaining systems and organs of the body and serves as the storage of ions and bone marrow [1,2]. As a dynamic tissue, bone is in a constant remodelling process to adapt to the mechanical demands and to repair small lesions that may occur [3]. Nonetheless, the presence of a critical size bone defect results in delayed unions or non-unions, which negatively affect the restoration of bone function [4].

One of the causes of the increase of life expectancy over the last decades is the extensive use of medical implants, among which bone substitutes are one of the most commonly used ones. Indeed, bone is the second most transplanted tissue worldwide, right after blood transfusions. The use of bone grafts for tissue repair and regeneration is not only due to the aging population, but can also be due to bone fractures, tumour resection, and bone diseases [5]. Autologous grafts are the strategy used in the majority of cases since they contain all the elements essential for bone regeneration (osteogenic cells, osteoinductive growth factors, and a matrix that supports bone adhesion and growth), and are collected in the individual, which minimize the risks of infection. However, their availability is limited and there is a risk of donor site morbidity [6,7]. Allografts emerge as an alternative to overcome the drawbacks associated with harvesting the autografts; however, they are associated with risk of infection and a high non-union rate with host tissue [6,8]. Thus, bone tissue engineering has been proposed as a promising alternative to the current bone grafting approaches. Even before Christ, several metals such as bronze and copper were used for the union of fractures. It was this type of thinking that persuaded several heath care professionals to try to introduce foreign materials into the bone tissue to compensate for bone defects and restore function [9].

A biomaterial may be defined “as any substance (other than drugs) or a combination of substances of synthetic or natural origin, which may be used for any period of time, as a whole or as part of a system which treats, tissue, organ or function of the organism”. The performance and success of biomaterials are dependent on the interactions that occur at their interface with the organism, which, in turn, are intrinsically related to the compositional and morphological properties of the material, in addition to the health status and daily activities of the individual in whom the biomaterial is inserted. All these features determine the biocompatibility of the material, which assumes that the implanted biomaterial does not cause adverse reactions that are either toxic or carcinogenic to the individual in which it is applied [10,11].

The biomaterials employed for bone tissue engineering are mainly divided into two main groups: ceramics or polymeric materials. Each of these materials has specific advantages and disadvantages [12]. The close resemblance between the chemical composition of the inorganic part of the bone and hydroxyapatite (HA-Ca_10_(PO_4_)_6_(OH)_2_, almost bioinert) and tricalcium phosphate (TCP-Ca_3_(PO_4_)_2_, resorbable) stimulated intensive research efforts to develop synthetic calcium phosphates (CaP) based bone grafts, including pure and biphasic compositions [10,11]. The discovery of the bone bonding ability of bioactive glasses through the formation of a layer of HA when exposed to physiological fluid [13] was an important milestone in the development of synthetic bone grafts. Nevertheless, these materials are brittle and exhibited unpredictable dissolution rates [14]. On the other hand, synthetic polymers have been developed with tailored characteristics; however, due to their lower bioactivity, they can be associated with a risk of rejection. Natural polymers, despite being biocompatible and enhancing cell adhesion and differentiation, have a limited supply and they exhibit poor mechanical properties and immunogenicity [15]. Bone is a composite material with an inorganic phase, HA, and an organic phase, mainly composed of collagen. Thus, the ceramic-polymer composites strategy has been widely explored by combining two different materials, thus overcoming the drawbacks of the individualised components [14,15,16,17,18].

The bone tissue engineering strategy involves the use of porous three-dimensional (3D) scaffolds that act as temporary supports and provide a suitable environment and architecture for bone regeneration and development [19]. Scaffolds for bone tissue engineering should have an interconnected porous structure and be highly porous with an adequate size for allowing cell adhesion and proliferation and also to ensure the diffusion of oxygen and nutrients to the cells and the removal of waste products. Moreover, the degradation rate should be similar to the formation of new tissue and the mechanical properties should match those of bone [12]. With the conventional techniques used to obtain porous scaffolds, it is difficult to have satisfactory control over the pore size, geometry, spatial distribution, and interconnectivity. These obstacles can be overcome using solid freeform fabrication techniques like robocasting, which enables precise control over the internal scaffold structure to be exerted [20]. Over the past years, significant efforts have been made towards developing scaffolds for bone regeneration, the development of CaP scaffolds, the preparation of composite scaffolds, and the incorporation of cells and growth factors into the scaffolds.

On the other hand, natural marine materials, like corals or cuttlefish bone (CB), which possess unique and very attractive architectures, have been studied as potential materials to support bone growth [21,22]. Regarding corals, the research has mainly focused on their partial or total transformation and their posterior combination with mesenchymal stem cells (MSCs) and/or growth factors. On the other hand, CB has been explored as a biomaterial in its natural form, but also after being transformed into CaP materials, with or without the incorporation of polymers to obtain composite materials with enhanced mechanical integrity.

## 2. Bone Tissue

Bone is a specialized, mineralized, and vascularized connective tissue that, along with cartilage, forms the skeletal system. Physically, bone acts as a support and site of muscle attachment for locomotion. The bone rigidity and hardness originating from the deposition of minerals such as CaP and carbonate inside the organic matrix in an organized functional way allows the skeleton to maintain the shape of the body and the protection of vital organs and bone marrow. In addition, the bone physiological functions include hematopoiesis, a process by which blood cells are formed, and mineral homeostasis, a reservoir for calcium, phosphate, sodium, potassium, zinc, and magnesium [1,23].

### 2.1. Bone Morphology

**Figure 1:**
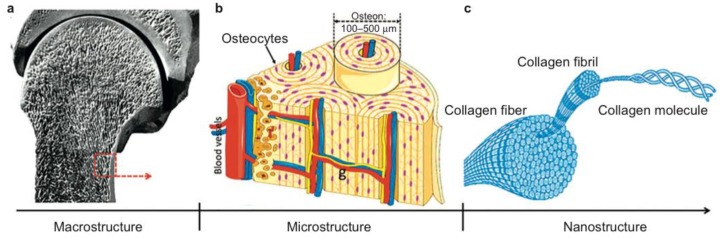
Hierarchical organization of bone tissue: (**a**) Macrostructure of cortical (dense shell) and cancellous bone (porous bone at both ends); (**b**) Microstructures of the osteons (20–30 concentric layers of collagen fibres, called lamellae) and trabeculae; (**c**) Nanostructures of collagen fibrils, which form the collagen fibres. The HA crystals are embedded in these gaps between collagen molecules and increase the rigidity of the bone [23].

Bone tissue is composed of two main parts: a compact shell called cortical bone and a porous core known as cancellous bone [2]. Cortical bone forms the outer wall of all bones and, in the adult human skeleton, represents 80% of the skeletal mass. It is composed of repeating osteon units and, the majority of it is calcified (80 to 90% of volume). It is associated with mechanical properties and, thereby, responsible for the supportive and protective function of the skeleton. The remaining 20% of the bone is cancellous bone, made of an interconnecting framework of trabeculae where only 15 to 25% of the volume is calcified and the empty spaces that are usually filled with bone marrow are the source of undifferentiated cells. It is mainly associated with metabolic functions, but also plays a role in biomechanical functions [1,2,23]. The degree of porosity of cancellous bone contrasts with the denser structure of cortical bone irrigated by a series of fine channels filled with blood vessels. The outer and inner bone surfaces are covered by the periosteum and the endosteum membranes, respectively, which play important roles in the nutrition of the bone tissue and supply of osteoprogenitor cells, which divide by mitosis and differentiate into bone forming cells, osteoblasts, and osteoclasts for bone formation and repair [2]. Additionally, the trabeculae and osteon units are composed of collagen and CaP crystals. The collagen fibrils include a 67 nm periodicity and 40 nm gaps between collagen molecules. The CaP crystals are embedded in these gaps between collagen molecules and increase the rigidity of the bone (Figure 1) [23].

### 2.2. Bone Composition

Bone tissue consists of a bone matrix (~90%) and bone cell populations (~10%), namely osteoblasts, osteoclasts, osteocytes, and bone lining cells [2]. The bone matrix is a composite material consisting of 65% mineral phase, HA, and 35% organic phase (~90% type I collagen (ColI), ~5% noncollagenous proteins, ~2% lipids by weight), in addition to a residual amount of water [24,25].

#### 2.2.1. Mineral Phase of Bone

Bone mineral consists of carbonated HA that is thin (1.5–4 nm) plate- or needle-shaped, incorporated within collagen fibrils, and orientated with the c-axis in the direction of the fibril [26,27]. The inorganic matrix material essentially consists of a carbonated calcium deficient HA (CDHA) [2]. The carbonate ions (CO_3_^2−^) might be incorporated into the HA lattice, partially substituting the OH^−^ sites or the PO_4_^3−^ groups to form A-type HA or B-type HA, respectively, or AB-type HA when both substitutions occur concomitantly [6]. But several other ionic impurities can be found in the inorganic matrix of biological HA, including small additions of citrate, fluorine, chorine, sodium, potassium, magnesium, strontium, zinc, iron, etc., incorporated in the crystalline lattice or adsorbed by its surface [2].

#### 2.2.2. Organic Phase of Bone

The organic phase of bone is mainly composed of ColI; nonetheless, other proteins, the so-called noncollagenous proteins, account for ~5% of the total bone weight [28].

ColI is essential in the bone since it provides elasticity to the tissue, stabilizes the extracellular matrix (ECM), acts as a template for initial mineral deposition, and binds to other macromolecules [28]. This molecule consists of a unique triple helical molecule, forming spaces within the collagen fibrils [29]. Additionally, these spaces are aligned to form thin and extended grooves where the intrafibrillar crystals form, thereby limiting the possible primary growth of mineral crystals, forcing them to be discrete and discontinuous [26,30]. Although ColI is the most abundant protein in mature bone, other collagen types, including types III, V, and VI, are also present in the bone [26].

Bone is also composed by non-collagenous proteins. Most of these proteins are not exclusive to bone; however, some of them, such as osteocalcin (OC), osteonectin (ON), osteopontin (OPN), and alkaline phosphatase, play fundamental roles in bone [25,26,28]. Generally, some of these proteins play a structural and mechanical role and other non-collagenous proteins modulate the functions of different bone cells by interacting with their cell-surface receptors, proteases, hormones, and other biomolecules, including proteoglycans and collagens. Particularly, OC and OPN are important for fracture resistance, and in older osteonal bone, their concentrations are lower [31]. Additionally, these proteins can also regulate collagen fibril mineralization and modulate cell division, migration, differentiation, and maturation [25].

#### 2.2.3. Bone Cells

##### Osteoblasts

**Figure 2:**
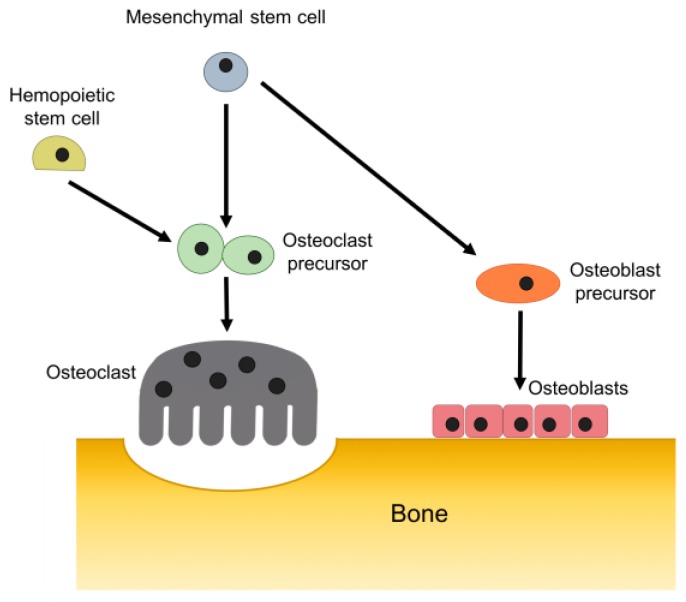
Evolution of osteoblasts and osteoclasts of bone.

Osteoblasts, which represent 4–6% of the total bone cells, are cuboidal cells that are accommodated in clusters along the bone surface and, therefore, do not function individually. These cells are derived from osteoprogenitor mesenchymal stem cells of the bone marrow stroma, which are multipotent adult cells that can differentiate into specialized cells including osteoblasts, chondrocytes, and adipocytes when appropriate stimuli are applied [32,33,34,35]. Osteoblasts are responsible for the synthesis of the bone matrix and subsequent mineralization. These cells are also responsible for the synthesis of the organic matrix and regulation of calcium and phosphate fluxes [2]. Under a variety of stimuli, osteoblasts can produce diverse growth factors including insulin-like growth factors (IGF), platelet-derived growth factors (PDGF), basic fibroblast growth factors (bFGF), transforming growth factor-beta (TGF-β), and bone morphogenetic proteins (BMPs) [3,36]. The production of the bone matrix by osteoblasts (Figure 2) occurs in three successive phases: the production and maturation of the osteoid matrix and its subsequent mineralization. Firstly, osteoblasts secrete collagen proteins, mainly ColI, and non-collagenous proteins and proteoglycan which form the organic matrix. Thereafter, mineralization takes place in vesicular and fibrillar phases [3,36,37].

##### Osteoclasts

Osteoclasts, responsible for bone resorption, are giant multinucleated cells containing one to more than 50 nuclei [1,2,3,36]. These cells are derived from mononuclear cells of the hematopoietic stem cells lineage, under the influence of a variety of factors (Figure 2) [38]. These cells are normally found in contact with a calcified bone surface and within a lacuna (Howship’s lacunae) due to its own resorptive activity. Osteoclasts bind to the bone surface through a process that involves the binding of integrins expressed in the osteoclast with specific amino acid sequences within proteins at the surface of the bone matrix [3]. Osteoclasts resorb bone by acidification and proteolysis of the bone matrix and HA crystals encapsulated within the sealing zone. Firstly, the process involves the mobilization of the HA crystals through the digestion of their link to collagen. Afterwards, the residual collagen fibers are digested and the residues are either internalized or transported across the cells and released at the basolateral domain [3,39].

##### Osteocytes

Osteoblasts have the capacity to secrete bone matrix during the differentiation process and some of them are immobilized and involved in their own bone matrix and give rise to osteocytes [40,41]. These are the most abundant cells in bone, representing 90–95% of the total bone cells [36]. These cells are responsible for the detection of microfractures and, thereby, play a crucial role in bone remodelling through the regulation of osteoclast and osteoblast activity, functioning as an endocrine cell [41]. Nevertheless, the functional activity of the osteocytes is modified with cell age. On one hand, a young osteocyte has most of the structural characteristics of the osteoblast and a low cell volume and capacity of protein synthesis. On the other hand, an older osteocyte, which is located deeper within the calcified bone, presents with a further decrease in cell volume and an accumulation of glycogen in the cytoplasm. Lastly, the osteocytes are phagocytosed and digested during osteoclastic bone resorption [41].

##### Bone Lining Cells

Bone lining cells are flattened osteoblasts derived from the bone matrix and their function is to control the flow of minerals between the bone tissue and the extracellular fluid and the coordination between bone resorption and formation [42].

### 2.3. Bone Remodeling

Bone is a dynamic tissue that undergoes constant remodelling through life. It possesses a unique self-regeneration capacity, which, in many cases, enables bone injuries and fractures to heal without scar formation by responding to metabolic needs and adapting to the mechanical stresses applied to the tissue. This process is fundamental to maintaining an adequate bone mass, appropriate mechanical properties, and the integrity of the skeleton [3,42,43,44].

**Figure 3:**
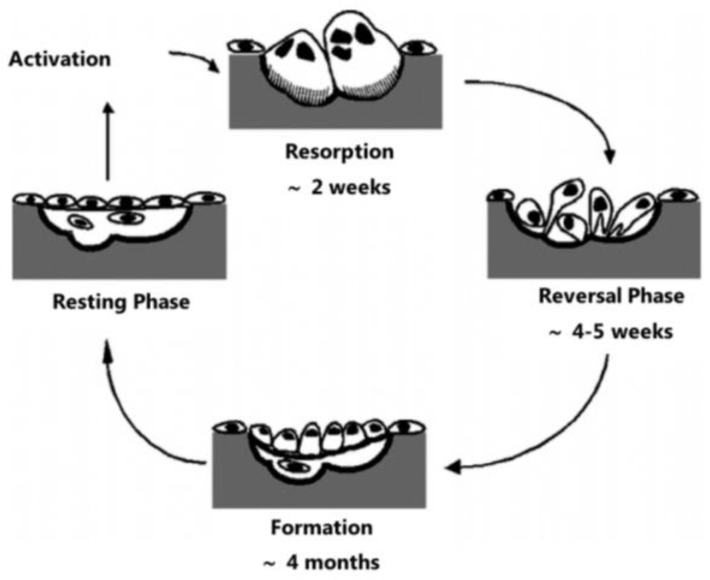
Bone remodelling cycle. Adapted from [1].

The remodelling is a consequence of a synchronized action of osteoclasts and osteoblast cells. The cellular activity at the remodelling site is characterized by four consecutives phases that transform a resting surface into a remodelling zone: activation, resorption, reversal, and formation (Figure 3). The activation phase consists of the recognition of the lesion suffered or the mechanical requests of the osteocytes. This activation will cause the retraction of bone lining cells and the recruitment of osteoclast cells through the release of cytokines [32]. Resorption begins upon a signal that leads to the migration of partly differentiated mononuclear preosteoclasts to the bone surface, which consequently form multinucleated osteoclasts. This process is characterized by a demineralization of the bone matrix, from a process of acidification of the zone to being adsorbed, dissolving crystals of HA, and degradation of the organic part of the bone by the action of proteolytic enzymes, leading to the formation of gaps. After the resorption phase, the reversal phase takes place. During this phase, mononuclear cells prepare the bone surface for bone formation and provide signals for the recruitment of osteoblast precursor cells that will proliferate and differentiate. Once the reversal phase is completed, bone formation takes place, where osteoblasts lay down until the resorbed bone is fully replaced by new bone [3,32,42]. The formation of bone tissue occurs in two stages: bone matrix production and its mineralization. Initially, osteoblasts synthesize the osteoid that will function as a support for the deposition of the mineral phase [2]. Mineral deposition occurs between the collagen fibers, due to the conformation of the collagen molecule that acts as a nucleation agent for HA that precipitates as mineral nodules [45]. When bone formation is completed, the osteoblasts can undergo apoptosis or terminal differentiation in osteocytes or bone lining cells, concluding the remodelling process [42]. A prolonged resting period begins and lasts until the beginning of a new remodelling cycle [3].

Importantly, in homeostatic equilibrium, bone resorption and bone formation take place in a coordinated way and, therefore, old bone is replaced by new tissue adapting to the mechanical load and strain. This homeostatic equilibrium only occurs during the first three decades. Indeed, it is precisely in the third decade when bone mass is at the maximum, and this is maintained with small variations until the age of 50. Posteriorly, resorption predominates and bone mass starts to decrease [3,40,43].

## 3. Bone Grafts

### 3.1. Evolution of Life Expectancy and the Need of Bone Grafts

The human life expectancy at birth has remarkably increased, especially since the mid-1800s, and has continued to rise during the following century [46]. There has been an impressive gain of about 30 years in life expectancy in western Europe, the USA, Canada, Australia, and New Zealand—and even larger gains in Japan and some western European countries, such as Spain and Italy. This trend is consensually attributed to a complex interplay of advances in medicine and public health, coupled with new organization modes at different societal levels, including economic-, political-, and behavioral-related changes [47]. Indeed, improvements in medicine are often pointed out as the most impacting factor responsible for the gains in human life expectancy [48]. However, it is worth mentioning that the earlier industrial revolution and the innovations in agricultural production and distribution enabled nutritional diversity and consistency for large numbers of people and are also seen as other important driving factors for lowering mortality [45]. In this regard, the former infectious leading causes of death (infectious and parasitic diseases) were gradually replaced by degenerative diseases such as cancers and diseases of the circulatory system, dramatically decreasing the risk of dying at earlier ages and postponing death to old ages [49].

The fantastic improvements in human life expectancy, behavioural nutrition, and physical activity came together with serious consequences in the function of the entire human musculoskeletal system, including: (i) the loss of bone mass or density due to decreasing contents of calcium and other minerals by osteoporosis, resulting in the increased brittleness and breakage of bone. This problem is commonly observed with a higher incidence in women after the menopause; (ii) increased incidence of degenerative diseases such as osteoarthritis in which the joints become stiffer and less flexible and the cartilage may begin to rub together and wear away concomitantly with a decrease of the synovial fluid in the joints and the eventual deposition of minerals (calcification). This bone joints breakdown may also lead to inflammation, pain, stiffness, and deformity. There is also an increased incidence of bone tumor resections; (iii) the decrease of the cushioning effect exerted by the gel-like invertebral disks in the spine because they gradually lose fluid and become thinner, resulting in shrinkage of the trunk and in a consequent overall height decrease; (iv) increased rate of teeth loss or extraction and the need to maintain a dense and healthy jaw through bone augmentation to prevent its natural deterioration; (v) increased incidence of serious trauma injuries associated with lifestyle changes (participation in sports); and (vi) the refusal of the public to tolerate the slightest limitation in mobility.

The presence of these pathological conditions (critical-size defects) is intrinsically related to an insufficient blood supply which impairs proper revascularization and negatively influences bone differentiation. Together, these events result in delayed unions or non-unions. As a consequence, there is a requirement for the bone grafts substitutes, that when accepted by the body provide a suitable framework for the growth of new living bone [50,51]. As the native bone grows, it gradually replaces the graft material, resulting in a fully integrated region of new bone.

### 3.2. Ideal Bone Graft

An ideal bone graft should provide osteointegration, osteconduction, osteoinduction, and osteogenesis [52]. Osteointegration is the ability to have a structural and functional connection between the living bone and the surface of the graft, and in this way, the formation of fibrous tissue does not take place at the bone-implant interface [52,53,54]. Osteoconduction is a characteristic by which bone grows on the surface of the graft [52,53]. Osteoinduction, is the ability of primitive, undifferentiated, and pluripotent cells from the surrounding host tissue to develop osteoprogenitor cells followed by the production of osteoblasts [51,52,53]. Osteogenesis is the capacity to produce new bone by osteoblasts present within the bone graft [51,52].

The most common sources of bone grafts include autografts, allografts, xenografts, and natural or synthetic bone graft substitutes [55]. These different bone grafts and their respective virtues and disadvantages are described in the following section.

### 3.3. Autografts

Autografts remain as the gold standard, constituting approximately 58% of bone grafts [5,51,56]. They can be harvested from non-essential bones of the patient, such as the iliac crest (the most common source), fibula, or metaphyses of long bones. Autografts for dental procedures are typically harvested from the jaw, hard palate, or the chin. If there is not enough bone available in these areas, the tissue graft may be taken from the hip or shinbone [7,56,57]. Autografts are known to be an optimal option with osteoinductive, osteogenic, and osteoconductive properties. Fresh autografts contain all the elements essential for bone regeneration, like the preservation of the trabecular architecture, and the presence of viable cells and growth factors (BMP-2 and -7, bFGF, IGF, and PDGF). In this way, autografts are rapidly incorporated into the host site which lacks immunogenicity [7,51,55]. The periosteum and nutrient artery are generally harvested with the piece of autologous bone and blood vessels that can be anastomosed to the blood vessel at the recipient site. Once the transplanted bone is secured into its new location, it generally restores the blood supply to the bone in which it has been attached. Despite the advantages, the use of autografts implies additional surgery, with a donor-site morbidity related to blood loss, wound complications, local sensory loss, and chronic pain [51,52]. Besides the limited availability, autografts require additional surgical time and costs. Therefore, other bone graft options should be considered [51,52,55,56].

### 3.4. Allografts

The drawbacks associated with the use of autografts can be overcome by the use of allografts, which in turn represent about 34% of bone substitutes [56]. Allografts are derived from humans, like autografts; however, they are harvested from an individual other than the one receiving the graft [51]. Different sources can be used, and they can be harvested from cadavers of human individual donors who donate their bodies for the benefit of science. A multi-organ donor is another source of bone, which is associated with the long bones acquired under a sterile condition in the operating theatre after organ explantation. They can also be sourced from people who are in need of repairing and regenerating bone defects and in which the most common source is the femoral head of a patient undergoing a hip replacement. Before the use of any allograft, the donor must be thoroughly screened to ensure that no infectious diseases are present. Nevertheless, there is always a risk of the transmission of an undetected viral or bacterial disease [8,58]. Allografts can be applied as structural forms or as bone chips. Additionally, they can be processed as mineralized or demineralized, fresh, fresh-frozen, or fresh-dried forms. Allografts have osteoinductive and osteoconductive properties; however, they also have lower osteogenic potential due to the absence of viable cells [8,51,55]. Complications linked with allografts include infections, a high non-union rate with host tissue, and fracture [51,52].

### 3.5. Xenografts

Xenografts are another alternative to autografts. This type of bone graft is harvested from non-human species, commonly from pigs, cows, and horses. The first bone graft procedure dating from 1668 was allegedly performed by the Dutch surgeon Job Van Meekeren, who harvested a bone derived from a dog’s cranium and implanted it in a soldier’s skull to successfully repair a traumatic defect [59]. In this regard, due to the large quantity of donors, xenografts may be more readily available and less expensive. Nevertheless, the potential transmission of bioactive material that causes diseases or rejection in the host remains as a threat. Many studies have attempted to investigate protocols that might be suitable for eliminating bioactive components such as heterologous cells, xeno-antigens, and DNA material, while preserving those that are essential for the proper functioning of ECM such as collagen helical macromolecules and water absorbing proteoglycans [60,61]. The adverse consequences of the decellularization process on the natural organization and physiological functionality of tissues intended as bio-inspired scaffolds have been addressed by several authors [62,63,64,65,66]. In order to avoid unfavourable immune responses, xenografts require a more sterile process, which can result in a loss of osteogenic and partly osteoinductive properties [51,67]. An alternative approach to prepare safe xenografts for bone regeneration involves chemical and thermal treatments in order to remove all the organic substances from fresh animal bones. Sang-Hoon Rhee, et al. [68] patented a method for preparing safe bovine-derived hone graft substitutes, which do not have the risk of infection with bovine spongiform encephalopathy. The method comprises treating bovine bone with sodium hypochlorite, followed by a heat treatment at temperatures within the range of 600–1000 °C. The organic substances are burned out and the resulting bone graft substitute does not cause an immune response. The patent is associated with a commercial product (Geistlich Bio-Oss^®^, Switzerland), which has allegedly been documented in more than 900 publications [69,70]. Other similar porcine- and bovine-derived commercial bone graft materials are available. Some examples are the products under the trade names of Symbios^®^, Switzerland, Endobone^®^, United States of America, and Straumann^®^ XenoGraft, Switzerland [71].

Despite this, several concerns still persist in relation to the use of bone grafts to face all the health problems derived from aging, trauma, and degenerative diseases, leaving room for an increasing search for alternative synthetic bone substitutes [72].

## 4. Bone Tissue Engineering

The shortcomings of bone grafts and the need to face all the health problems originating from aging, trauma, and degenerative diseases are the main driving forces for developing new synthetic biomaterials. Other stimulating reasons for the recent boom in the development of new synthetic biomaterials and implantation devices include: (i) increasing awareness among patients and doctors of the numerous co-morbidities associated with autograft harvesting; (ii) the elevated regulatory scrutiny and recalls imposed on allograft tissue banks for distributing human bone and soft tissue products that were improperly screened for infectious diseases; and (iii) the recent developments in surgical procedures and materials that allow new procedures to be available. Accordingly, a wide variety of synthetic bone substitutes have been developed and employed over the past 50 years, contributing to the actual market trend for shifting away from autografts to bone graft substitutes, and from cadaveric allografts to synthetics [71].

Bone tissue engineering is a multidisciplinary field that applies the knowledge of bioengineering, biology, cell transplantation, and material science to create new biomaterials that will interact with biological systems to treat, strengthen, and, thereby, regenerate damage tissues and restore their function, instead of replacing them [12,73]. Normally, it involves the use of porous 3D scaffolds that, along with cells and bioactive factors, provide structural support for cells to spread, migrate, multiply, and differentiate, and for new tissue to be formed [12,74]. In this way, scaffolds act as a temporary ECM inducing the natural processes of tissue regeneration and development [75].

### 4.1. Biomaterials for Bone Tissue Engineering

Biomaterials have evolved through three generations. The first generation of biomaterials was developed during the 1960s and 1970s and was intended to provide adequate functional properties without causing harmful effects on the host. This generation of biomaterials was not specifically developed for medical use and consisted of widely available industrial materials like elastomeric polymer and silicon rubbers. Their selection was based on their bioinertness and suitable physical properties. With the emergence of the first generation of biomaterials, tens of millions of individuals had their quality of life improved in 20 years. The goal of the second generation of biomaterials was to shift from a completely bioinert reaction to the production of materials that could induce a controlled reaction with the host tissue in order to have a therapeutic effect and hence have a bioactive behaviour. This generation of materials is associated with the development of resorbable biomaterials in which the degradation rates could be adjusted to the desired applications. In this way, the implanted material could be degraded into a soluble and non-toxic product by the host, consequently eliminating the interface between the implanted site and the host tissue. These materials are in clinical use in fields like orthopaedic and dental surgeries, in localized drug release applications, and in cardiac assist devices. The third generation of biomaterials has the objective to support and stimulate the regeneration of functional tissue, thereby inducing cellular responses at the molecular level by using two routes, tissue engineering and in situ tissue engineering. These approaches involve the use of scaffolds, cells, and growth factors and have already been responsible for the successful replacements of damaged skin, cartilage, bladders, corneal epithelium, and trachea [11,76,77].

The field of biomaterials for bone tissue engineering is constantly evolving. These biomaterials can be subdivided into organic polymers, which can be subdivided according to their synthetic or natural source, and inorganic materials, such as CaP and bioactive glasses. By combining the former materials, it is possible to form composites [12,78,79].

#### 4.1.1. Organic Materials

Polymer materials are extensively used in tissue engineering. They can be divided into natural and synthetic polymers.

##### Natural Polymers

Natural polymers constitute the native ECM and, thereby, have an excellent biocompatibility and a low immunogenic potential. They are bioactive as they have the capacity to interact with the host’s tissue. Their structure is organized and comprises ligands that can be bound to cell receptors [18,80,81]. Although, in some cases (e.g., chitosan and starch), their source is almost unlimited, most of them lack a high enough quantity. These polymers are difficult to process and their degradation behaviours change from patient to patient since degradation involves enzymatic processes [81].

Collagen is the main component of ECM and, therefore, has an inherent biocompatibility, non-cytotoxicity, and non-inflammatory reaction. It has functional groups that improve cell adhesion and proliferation. This natural polymer, despite having a lower mechanical property, has a stable structure related to covalent cross-linking among the collagen fibrils. Moreover, it can be processed into different forms, powders, sponges foams, sheets, fibres, membranes, films, and injectable viscous solutions. However, collagen has a bath-to-bath variation in terms of physicochemical and degradation properties and is associated with a relative risk of infection [80,81]. Gelatin is a denatured protein of collagen and, thereby, has a relatively low antigenicity. It is a biocompatible and bioresorbable polymer with arginine-glycine-aspartic (RGD) sequences in its structure, the recognition sites of integrins, which mediate cell-cell and cell-ECM interactions and, consequently, improve cell adhesion. Moreover, its functional groups are more accessible, enabling chemical modifications. Despite all the advantages, gelatin has a limited mechanical strength and, normally, is combined with other polymers or ceramic materials to improve the mechanical properties [82].

Chitosan, the deacetylated chitin derivative, is a natural polymer with interesting properties for biomedical applications due to its biocompatibility, biodegradability, low toxicity, non-immunogenicity, and intrinsic anti-bacterial nature. This polymer enables cell adhesion and proliferation and, in addition, supports the formation of the bone matrix. As collagen, it can be processed into different forms. The degree of degradation can be adjusted regarding the degree of deacetylation [80,81].

Alginate, a polymer obtained from brown algae, has been widely used in the biomedical field. It has an exceptional biocompatibility, biodegradability, and non-toxicity. It is not an expensive polymer and is available in abundance. Alginate is a polymer that that can be easily modified, and, for instance, alginate gels produced through cross-linking with calcium, can be introduced through a minimal invasive procedure. Some drawbacks are associated with alginate like the slow degradation rate and inappropriate mechanical integrity that precludes long-term biomaterial implants.

Hyaluronic acid, obtained from the ECM of all connective tissue, exhibits good biocompatibility, immunoneutrality, and viscoelasticity [80,81]. It is beneficial for bone regeneration [83].

##### Synthetic Polymers

Synthetic polymers can be obtained under controlled conditions and, thereby, have a predictable batch-to-batch uniformity with reproducible and adjustable physicochemical properties (e.g., mechanical behaviour and degradation rate). Synthetic polymers enable tailoring the shape, porosity, and pore size, and incorporating chemical functional groups that improve tissue growth [80]. Normally, the degradation of synthetic polymers occurs through a hydrolysis process and, in this way, it does not vary between hosts [84]. In some cases, synthetic polymers are combined with natural polymers to improve cellular adhesion due to the presence of ligands that can bind to cell receptors in natural polymers [85,86].

Polyesters are the most used polymers in the field of bone tissue engineering and include poly(glycolic acid) (PGA), poly(lactic acid) (PLA), poly(lactic-co-glycolide (PLGA) (which is a copolymer of PGA and PLA), and poly(ε-caprolactone) (PCL). Polyesters can be dissolved in organic solvents, with the exception of PGA, which is only soluble in highly fluorinated solvents due to its highly crystalline structure. These polymers are biocompatible and the by-products originating from degradation are glycolic acid and lactic acid, which are natural metabolites and, therefore, not harmful to the human body. Nevertheless, these by-products can reduce the local pH and, consequently, may induce an inflammatory reaction [80,81]. PGA has a short degradation period (from four to 12 months) and high values of tensile strength and modulus of elasticity. On the other hand, due to its hydrophobicity, the degradation rate of PLA is between 12 months and two years. The low values of PLA tensile strength and modulus of elasticity can be improved by the use of copolymers of lactic acid and glycolic acid like PLGA [81,84]. PCL has a degradation rate slower than the other polyesters, and can reach 24 months. An improvement of this degradation rate can be achieved by a co-polymerization process. Additionally, PCL is biocompatible and exhibits suitable mechanical properties for bone tissue engineering. 

In comparison with polyesters, polyamides exhibit better mechanical and thermal behaviour, but they take a long time to degrade in the human body. In this regard, poly(ester amide)s have received particular attention, since they can combine the biodegradability of polyesters and the mechanical and thermal behaviour of polyamides [87,88]. Alternatively, poly(ester urea)s have been explored in the field of tissue engineering since they are non-toxic and the degradation rate and the mechanical properties can be adjusted. Moreover, the versatility in the functionalization of these polymers can be beneficial in designing a scaffold for bone tissue engineering [89,90].

Poly(propylene fumarate) (PPF) is an unsaturated linear polyester that, upon degradation, forms propylene glycol and fumaric acid, products that are biocompatible and easily removed from the body. The mechanical properties can be improved via cross-linking through the fumarate double bond or via thermal or photo cross-linking through the active double carbon chain, and in this case, also the degradation rate can be tailored [80,81]. PPF is in liquid form before cross-linking, which allows it to be injectable and, consequently, to be suitable for orthopaedic implants in minimal invasive procedures.

Polyanhydrides are biocompatible degradable polymers with good properties of drug-controlled release. Nevertheless, they lack appropriate mechanical properties for load-bearing applications. To overcome this drawback, polyanhydrides are copolymerized with polyamides with surface-eroding properties [80,81] or alternatively become photo cross-linkable and injectable [81].

Polyurethanes started to be particularly explored as a biomaterial for bone tissue engineering in the past two decades, since when adequately designed, these polymers enjoy a set of interesting properties, including non-cytotoxicity, biocompatibility, biodegradability, and the capacity to promote in vivo calcification. They can be applied as injectable void fillers, drug delivery systems, scaffolds, and shape memory materials due to their capacity to vary from hydrophobic to hydrophilic, and from rigid to flexible, or to exhibit thermoplastic to thermosetting behaviours [91].

#### 4.1.2. Inorganic Materials

CaP, such as β-TCP and HA, and bioactive glasses have been explored as bone substitute biomaterialsdue to their chemical and structural similarity to the mineral component of bones and teeth.

##### Calcium Phosphates

CaP materials (Table 1) can be found in various forms from thin coatings in metallic implants, improving their biocompatibility for temporary structures that are replaced by new bone. They can be produced in large quantities, with a relatively low-cost. In addition, CaP are stable and, therefore, are available off-the-shelf [27,92].

HA and β-TCP are two of the most used CaPs. HA is one of the most stable phases under physiological conditions and has a low solubility and, consequently, a slower resorption rate. Traditionally, HA is prepared in aqueous precipitation by mixing adequate quantities of Ca^2+^ and PO_4_^3−^ containing solutions at a pH above 9, followed by filtration, drying, and sintering. The as-synthesized HA is poorly crystalline and often non-stoichiometric. On the other hand, β-TCP is a high-temperature phase, which is only prepared at temperatures above 800 °C. However, it is important to note that, for temperatures approximately above 1125 °C, β-TCP transforms into a high-temperature phase α-TCP. When compared with HA, β-TCP is more soluble and has a lower mechanical stability [85,86]. Therefore, an optimum balance is often achieved by combining the more stable HA phase and a more soluble β-TCP phase to obtain biphasic CaP (BCP). This combination leads to materials with controlled bioactivity and a more appropriate balance between resorption/solubilisation, which guarantees the stability of the biomaterials while promoting bone ingrowth [87,88].

Since CaPs are similar to the mineral phase of bone, they are recognized as biocompatible, a material not foreign to the body, and also non-toxic. Importantly, CaPs exhibit a bioactive behaviour and are integrated into the body by processes that are equal to those of bone remodelling, leading to an intimate physicochemical bond between the biomaterial and bone. Moreover, CaPs are known to be osteoconductive and support cell adhesion and proliferation [93]. The main drawback associated with CaP biomaterials is their poor mechanical properties. Namely, their brittle nature, with a low fracture strength, represents a main concern in high load-bearing applications [27,79,92,93]. This brittle nature is associated with high-strength ionic bonds and can be manipulated by composition, crystallinity, grain size, and grain boundaries, as well as by porosity [92].

The crystalline structure of CaPs enables the incorporation of trace amounts of certain ions existing in bone composition [96]. It is recognized that the incorporated ions like strontium (Sr) [97,98,99,100], magnesium (Mg) [98,100], manganese (Mn) [7,99,100], or zinc (Zn) [101,102] in a single or combined manner, play fundamental roles in bone development. Sr is present in bone in considerable amounts and, in particular, at regions of elevated metabolic turnover [96,100]. Its presence is associated with the increase of osteoclast apoptosis and the enhancement of osteoblastic cell proliferation and collagen synthesis, which subsequently maintain bone formation and inhibit bone resorption [97,98]. Mg is related to the mineralization of calcified tissue, and its amount starts to be higher and then decreases during the calcification process. Further, this ion influences bone metabolism as it plays a role in osteoblast and osteoclast activity [96]. The incorporation of Mn exerts positive effects on bone growth as it promotes cell adhesion due to the fact that in the presence of Mn, there is an increase of the ligand-binding affinity of integrins, which are receptors that mediate the interaction between cells and the ECM and activate cell adhesion [100]. Zn, in a similar way as Mg, promotes bone formation and its deficiency in the body is associated with a decrease of bone density. Furthermore, Zn influences the crystallinity and morphology of biological apatite crystals [101,103].

#### 4.1.3. Composite Materials

Hard tissues, like bone, should be able to support the load and when compared to soft tissues, should be stiffer and stronger. Thus, in most cases, instead of polymers, ceramics and metals have gained more attention. Nevertheless, some important drawbacks are associated with these two materials. Ceramics are more brittle and, in some cases, stiffer than bone; and metals are considerable stiffer than the bone. On the other hand, despite being more ductile, polymers do not normally exhibit enough stiffness for bone graft applications [104].

With this in mind, composite materials have been widely explored in the bone tissue engineering field since they combine at least two different materials in order to obtain bone graft substitutes with improved functionalities in terms of mechanical and osteoconductive properties.

This section will be focused on composites that combine ceramics and polymers, and despite recognizing that the resulting composite scaffolds can be obtained by mixing ceramic powder with a polymer solution and using different manufacturing techniques [17,105,106], by depositing ceramics onto polymers [17,107,108], or by the deposition of polymers onto ceramics [17,109,110,111,112,113], only the last one will be reviewed here.

Motealled et al. [109] produced 45S5 bioglass scaffolds by robocasting and studied the effect of their coating with natural (gelatin, alginate, and chitosan) or synthetic (PCL or PLA) polymers on the mechanical and in vitro bioactivity and degradation behaviour. The chitosan coating was highlighted for its mechanical and biological properties. The incorporation of this polymer enabled mazimixing the compressive strength and toughness (strain energy density). An improved bioactivity of the 45S5 scaffolds, translated by an accelerated formation of an apatite surface layer, was also registered in the presence of the chitosan coating. In addition, there was a decrease in the degradation rate of the majority of the coatings, and a consequent positive impact on the evolution of their mechanical properties. Shi et al. [113] produced a β-TCP scaffold through the polymeric sponge replication method and aimed to improve the mechanical properties of the scaffolds by coating them with PCL. It was observed that PCL addition significantly improved the compressive and bending strength, with the highest value being registered for the scaffolds containing 40% of β-TCP and 5% of PCL. Furthermore, the presence of PCL did not compromise the osteoblast cells’ proliferation and differentiation. The reinforcement of BCP scaffolds with not only PCL, but also with HA particles of different sizes and morphologies, was studied by Roohani-Esfahani et al. [112]. The produced BCP scaffolds were subsequently coated with PCL and nano (needle shape) or micron HA. The PCL coating enhanced the compressive strength of BCP scaffolds from 0.1 ± 0.05 MPa to 0.29 ± 0.07 MPa and 2.1 ± 0.17 MPa when using micro or nano HA particles, respectively. Moreover, among all the scaffolds, those coated with needle-shaped HA and PCL exhibited the strongest osteoblast differentiation and the highest alkaline phosphatase (ALP) activity, and an upregulation of osteogenic gene expression, namely runt-related transcription factor 2 (Runx2), ColI, OC, and bone sialoprotein. Apart from the former advantages, the polymer coating on ceramic scaffolds has also been widely used for drug delivery systems. For instance, Li et al. [110] coated 45S5 scaffolds with a solution containing poly(3-hydroxybutyrate-*co*-3-hydroxyvalerate) (PHBV) and vancomycin, an antibiotic used for infections that occur during bone disease treatment. The authors observed that the polymer coating improved the compressive strength and mechanical stability, while not negatively affecting the in vitro bioactivity. A sustained and controlled drug release was observed in the coated scaffold (99.9% in six days), contrary to what was observed when the drug was directly adsorbed on the 45S5 scaffold (99.5% in three days). The thickness and structure of the polymer coating can be dependent on the texture of the scaffold material, particularly on the specific surface area (SSA). Canal et al. [111] studied the influence of the previous parameters on the Simvastatin acid (SVA) release, a component that despite being used for the cholesterol treatment, is also known to stimulate osteogenesis by the up-regulation of BMP-2 expression. In this regard, β-TCP and CDHA scaffolds were coated with polycaprolactone-co-polyethyleneglycol (PCL-co-PEG) loaded with SVA. A low pressure plasma process was used, allowing the coating of inner regions of the scaffolds up to a certain depth. The work was divided into two parts: firstly, CaP discs were used to characterize the polymeric layer; in the second part, the release profiles of SVA from the coated CaP scaffolds were studied. The polymer layer on the β-TCP was about two times thicker in comparison to that on the CDHA, with the difference being attributed to a much higher SSA of CDHA (~33 times). Although the plasma coating of the polymer loaded with SVA had been beneficial for controlled drug release in the case of CDHA scaffolds, the thicker polymer coating on β-TCP tended to hinder the SVA release. On CDHA scaffolds, 90 min of continuous wave plasma discharges treatment blocked the drug release in the first 1.5 h and allowed a slow diffusion over 11 days. Reducing the treatment to 20 min enabled the slow release process to start from the beginning of the experiment.

### 4.2. Scaffolds for Bone Tissue Engineering

A wide variety of biomaterials and manufacturing techniques have been explored to produce a scaffold able to regenerate the bone. When designing an ideal scaffold for bone tissue engineering, some important characteristics should be considered: (i) firstly, the scaffold must be biocompatible. It should have the ability to support cell adhesion and proliferation on the surface and through the scaffold, without any negative effect on the host tissue, which may lead to reduced healing or cause rejection by the body [114]; (ii) the underlying premise of bone tissue engineering is to allow the replacement of the implanted scaffold by the ECM over time. In this regard, the scaffolds must be biodegradable and preferably able to degrade at a similar rate to bone formation. The by-products originating from the degradation process should be non-toxic and removed from the body without interference with other organs [12]; (iii) the scaffolds must have a highly interconnected porosity for successful bone growth and vascularization. Nowadays, it is recognized that a hierarchical porosity from macro- to nanoporous is beneficial for the development of new bone tissue [56,115]. Scaffolds with a pore size between 200 and 350 µm have been revealed to be optimal for bone growth. This allows cell infiltration and, subsequently, the formation of ECM, as well as the diffusion of nutrients and oxygen and the removal of waste products [114]. The presence of micro and nano porosity plays a fundamental role in cell attachment, biomineralization, and in vivo osteointegration [115]; (iv) an ideal bone scaffold should have mechanical properties that match the host bone properties and should be strong enough to allow surgical handling during implantation. Importantly, bone scaffolds must maintain their integrity from the time of implantation to the end of the remodelling process. The healing rate significantly changes with age, which should be considered when designing the scaffolds. Fractures from young individuals normally heal in approximately six weeks and mechanical integrity returns one year after fracture; however, in elderly people, this bone regeneration slows down. Furthermore, it is known that the enhancement of mechanical properties often occurs with the detriment of highly interconnected porous scaffolds. An implanted scaffold with good in vitro potential is likely to fail in vivo if the vascularization capacity is insufficient. With this in mind, it is essential to maintain a balance between mechanical properties and porous structure upon designing a successful scaffold [12].

## 5. Scaffold Fabrication Techniques for Bone Tissue Engineering—Robocasting

As previously mentioned, several aspects should be taken into consideration when designing a scaffold for bone tissue engineering, namely the porosity at different dimensions to allow cell adhesion and proliferation, but also vascularization for subsequent bone growth. Mechanical properties are also fundamental to providing an adequate mechanical support for bone repair and regeneration. Having close control over the pore size, geometry, spatial distribution, and interconnectivity is difficult when using conventional fabrication techniques to produce porous scaffolds (e.g., freeze drying, chemical/gas foaming, melt, molding, phase separation, fiber meshing, supercritical-fluid technology, and solvent casting in combination with particulate leaching) [20,77,116]. These obstacles can be overcome using additive manufacturing (AM) techniques, which enable the production of scaffolds with precise control of the internal scaffold architecture, without the need for subsequent machining [20,79,116]. Solid freeform fabrication, rapid prototyping, and 3D printing are some of the AM techniques that have been reviewed in detail elsewhere [19,20]. AM technologies enable the creation of complex 3D layer-by-layer structures directly from a computer-aided design (CAD). Alternatively, AM technologies enable using data from computerized tomography or magnetic resonance image medical scans to create CAD models that are then converted into STL-files. By using these STL-files, it is possible to match the scaffold’s external shape to the damage tissue site [20,77,116]. Along with the important advantage previously mentioned, AM techniques do not require many process steps and include little manual interaction [77].

**Figure 4:**
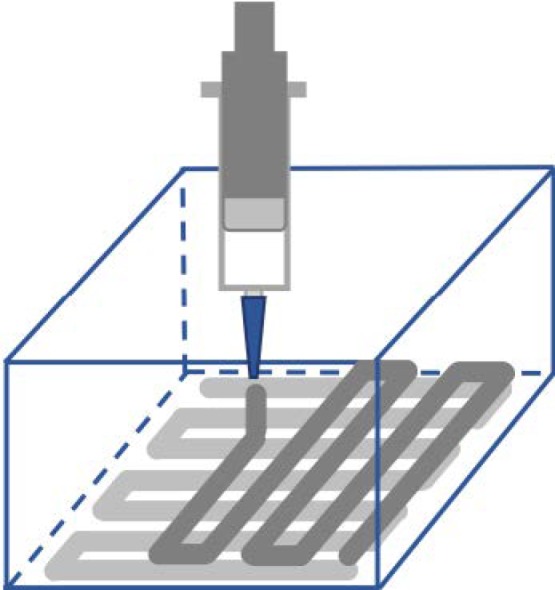
Schematic representation of the robocasting fabrication process. The ceramic scaffold is built layer-by-layer from a CAD model. The three-axis robotic arm moves the injection syringe while expressing the ceramic ink through the conical deposition nozzle to create the desired structure immersed in an oil bath.

Robocasting is one of the AM techniques that allows researchers to build scaffolds using a concentrated colloidal suspension (ink/paste) with neglible contents of organic additives without the need for a sacrificial supporting material [116,117]. Robotic deposition of a continuous filament capable of fully supporting their own weight takes place through a nozzle during the assembly layer-by-layer. To prevent non-uniform drying during assembly, during the fabrication process, the process of deposition is conducted within a non-wetting oil bath (Figure 4) [116,118,119].

The colloidal inks developed for robocasting require careful characterization and must satisfy two important criteria. Firstly, the ink must exhibit a well-controlled viscoelastic response so that it yields upon extrusion, but sets immediately upon deposition, to facilitate shape retention. Second, the ink must have a high colloidal volume fraction to minimize drying-induced shrinkage after assembly. In other words, the particle network must be able to resist compressive stress arising from capillary tension and, therefore, the extruded filaments can retain their shape across unsupported spans, and the subsequent layers do not cause the layers beneath to significantly yield and deform. These criteria require suitable control over the colloidal forces to first generate a highly concentrated stable suspension and then induce a dramatic change in the rheology of the system that promotes a fluid-to-gel transition [117,120].

Porosity in robocasting can be tailored on macro- (greater than 100 µm), micro- (1–30 µm), and submicron (less than 1 µm) scales. Macroscale porosity is introduced directly by the robocasting process as it draws successive layers. Robocast filaments arranged in latticed patterns create macroporous pathways in three dimensions. By varying the rod spacing and size, these pathways can be precisely constructed to produce highly uniform macropores. By incorporating porogen microspheres into the suspension prior to extrusion, it is possible to create micropores. With a suitable volume fraction of microspheres, the microporosity is interconnected. Lastly, the submicron porosity can be controlled by varying the temperature profile at which the produced scaffolds are sintered [120].

Due to all of the advantages of robocasting, this technique has been widely explored for the production of scaffolds in the field of bone tissue engineering. For instance, Miranda et al. [121] obtained β-TCP and HA scaffolds by robocasting. In order to achieve the ink for printing, the powder was firstly dispersed in distilled water containing a suitable amount of dispersant to obtain a highly concentrated suspension. Hydroxypropyl methylcellulose was subsequently added to increase the viscosity and an ink with the desired viscoelastic properties was obtained upon the addition of a flocculant agent like polyethylenimine. HA scaffolds were demonstrated to have a better compressive strength than the β-TCP scaffolds and this performance was emphasised upon the immersion for three weeks in simulated body fluid (SBF). Recognizing the advantages of a BCP material, Houmard et al. [119] reported the production of pure HA and two biphasic compositions (60HA/40 β-TCP and 20HA/80β-TCP) by robocasting using water-based Pluronic inks. The mechanical strength varied between 3 and 50 MPa according to the porosities, which varied from 80 to 25 vol.%, respectively, and also according to the rod size, in which thinner rods improved the mechanical properties. Furthermore, mechanical properties were also dependent on the composition, where HA exhibited the highest values and 60HA/40β-TCP the lowest. No evident degradation of the scaffolds in SBF or water was observed, despite a slight increase of Ca and P ions in water being recorded after five months. Using a similar procedure to obtain the ink for printing as Miranda et al. [121], Marques et al. [122] produced undoped and doped strontium and silver BCP scaffolds by robocasting. They studied different pore sizes and demonstrated that the mechanical properties were intrinsically correlated with the porosity fraction. Furthermore, the presence of doping elements improved the mechanical strength and cell proliferation and were effective in terms of the antimicrobial activity against *Staphylococcus aureus* and *Escherichia coli*. On the other hand, the incorporation of MSCs, as well as growth factors, into porous scaffolds enhanced bone formation and with this in mind, Del Rosario et al. [123] studied the effect of the incorporation of BMP-2, PDGF, and rat MSCs (rMSCs) in a β-TCP scaffold obtained by robocasting. BMP-2 was incapsulated in PLGA microspheres to allow a more sustained release and PDGF, in which the goal is to act in the first stages of bone repair, was crosslinked with alginate and formed a thin layer. The scaffolds exhibited a good cell viability and biocompatibility in vivo. A release of approximately 90% of BMP-2 and 80% of PDGF was observed after three weeks and two days, respectively. Nevertheless, there was no beneficial effect upon a dual delivery of BMP-2 and PDGF or upon the combination of BMP-2 and rMSCs. Aiming at producing composites from polymers and CaP, Hong et al. [124] and Maazouz et al. [125] developed PCL/HA and gelatine/HA scaffolds, respectively. Hong et al. [124] produced a composite scaffold with a PCL/HA ratio of 1. A lower cell adhesion on the composite scaffolds was observed when compared to a conventional cell seeding technique; nevertheless, the adhered cells remained viable. Moreover, it was demonstrated that the composite scaffolds stimulate osteogenic differentiation. Indeed, when compared with scaffolds of pure PCL, the PCL-HA scaffolds, there was an increase of ALP activity of rat bone marrow stromal cells (rBMSCs), as well as an up-regulation of the expression of bone-associated genes (ALP, ColI). α-TCP is a widely used material for the production of CaP cements that, when combined with water, leads to the formation of CDHA. Furthermore, the incorporation of gelatine enhanced the processing ability, and the mechanical and biological properties. With this in mind, and assuming that gelatine could ensure the viscoelastic properties for printing, Maazouz et al. [125] used a reactive ink that combined α-TCP with gelatine for the production of HA/gelatine scaffolds by robocasting. It was observed that gelatine was retained by chemical crosslinking. In order to allow enough time for printing, the setting reaction needed to be delayed. Upon setting, the scaffolds improved their compressive strength and a further increase was registered after the crosslinking reaction. The presence of gelatine improved cell adhesion and proliferation.

Some large bone grafts lack oxygen diffusion throughout the implant. The oxygen release is important in tissue survival during formation and can be achieved by the incorporation of peroxide into hydrophobic polymers, with a slow and sustained release of oxygen, or when incorporated into hydrophilic polymers upon undergoing rapid water absorption, originating from a faster polymer decomposition and oxygen generation. With this in mind, Touri et al. [126] used robocasting to produce BCP scaffolds that were posteriorly coated with a thin film of PCL, a hydrophobic polymer, in which different concentrations of calcium peroxide (CPO) were encapsulated. A sustained CPO release was observed. A coating with 3% CPO exhibited the best properties regarding osteoblast viability and proliferation under hypoxic conditions, which is a good indicator for bone growth.

Apart from CaP materials, bioactive glasses are also widely used in bone tissue engineering and have thereby been explored for the production of scaffolds by robocasting. Eqtesadi et al. [127] obtained 45S5 bioglass scaffolds via robocasting using a single processing additive, carboxymethyl cellulose (CMC). The scaffolds were sintered within the range of 500–1050 °C and for all of the temperatures, exhibited sufficient mechanical integrity and a compressive strength comparable to the one of cancellous bone. However, to mitigate the brittleness of these scaffolds and enhance the mechanical properties, they reinforced these scaffolds with graphene oxide (rGO) contents ranging from 0–3 vol.%, followed by sintering at 550 or 1000 °C. The mechanical properties were optimal for 1 vol.% of rGO reinforcement and a sintering temperature of 550 °C, for which there were enhancements in the fracture toughness and compressive strength of 850% and 290%, respectively [128]. Olhero et al. [129] used an alkali-free bioactive glass, FastOs^®^BG (Portugal), containing 70% diopside (CaMgSi_2_O_6_), 10% fluorapatite (Ca_5_(PO_4_)_3_F), and 20% tricalcium phosphate (3CaO·P_2_O_5_), to obtain porous scaffolds by robocasting. They obtained scaffolds with three different pore sizes (200, 300, and 500 µm) from pastes containing 47 vol.% solids using hydroxypropyl methylcellulose and Aristoflex^®^ TAC as binder and gelation agents, respectively. The measured compressive strength of the scaffolds was similar to the cancellous bone for all investigated pore sizes.

Zirconia-toughened alumina (ZTA) is a widely used material in hip arthroplasty because of its attractive mechanical properties. With this in mind, Stanciuc et al. [130] produced ZTA scaffolds by robocasting. They started from inks with solid loadings of 70 wt.% (~35.5 vol.%), which are too low for optimal processing. Shape retention during printing was only achieved by using an acidic water bath (deionized water and HCl; pH = 1.5) since it allowed ink coagulation. The authors reported that human primary osteoblasts were able to adhere onto the scaffolds and the microporous structure enhanced the expression of runx2 and ALP when compared to 2D-ZTA.

## 6. Marine-Derived Bioceramics as Scaffolds for Bone Tissue Engineering

Significant changes in the production of scaffolds have been introduced over the last years with the adoption of AM techniques and the noticeable increase of affordable AM bioprinters. Despite this, the development of technologies to facilitate the implementation of these bioprinters in regenerative medicine and clinical manufacturing is still the major identified roadblock. One of the most vital, but to date limiting, components required for the widespread adoption of bioprinting in regenerative medicine is the availability of effective bioinks.

Oceans are abundant sources of diverse materials with potential applications in healthcare, including, among others, bioceramics, biopolymers, fatty acids, toxins and pigments, nanoparticles, and adhesive materials [131]. In this regard, and in order to overcome the drawbacks associated with AM, marine skeletons, mainly composed of aragonite (CaCO_3_), have proved to be a promising alternative for bone tissue engineering taking advantage of their porous structure and mechanical strength [21,132].

Once cleaned, marine skeletons can be used as bone graft substitutes, either in aragonite or preferably after being hydrothermally transformed into CaP scaffolds, while keeping exactly the same porous architecture. The transformation can be partial or total, depending on the hydrothermal treatment conditions (temperature, time, chemical environment). The partial conversion of CaCO_3_ from marine exoskeletons into CaP means that the obtained products consist of composite materials with an inner calcium carbonate core and an outer layer with a composition close to that of the mineral part of the bone, making them viable bone grafts substitute materials [22,133,134,135,136]. The conversion from corals to porous HA was first performed by Roy and Linnehan in 1974 [133]. Since then, marine skeletons of cuttlefish [137], marine sponges skeletons [138], and nacre seashell [139] have been converted to HA, while maintaining their original structures, aimed at obtaining bone graft substitutes. In turn, sea urchin spines that consist of large crystals of Mg-rich calcite [(Ca,Mg)CO_3_] have been hydrothermally transformed into Mg-substituted TCP [140] that has been used as templates with an optimal range of pore size, channels, and structural network for bone growth.

The following sections of this review will be focused on the use of corals and CB as scaffolding systems in the area of biomedical applications.

### 6.1. Corals

Corals are marine invertebrates typically living in compact colonies of many identical individual polyps. Each polyp is a small sac-like animal that is only a few millimetres in diameter and a few centimetres in length, and has a set of tentacles surrounding a central mouth opening. Near the base, polyps absorb elements present in seawater, namely carbonic acid and calcium ions, and produce a calcium carbonate exoskeleton in an aragonite form, which grows over many generations. Apart from calcium carbonate, which represents 97–99%, corals also have oligoelements (0.5–1%), sodium (0.4–0.5%), magnesium (0.05–0.2%), amino acids (0.07%), and potassium (0.02–0.03%). Individual heads may grow by the asexual reproduction of polyps. But polyps also breed sexually by releasing gametes simultaneously over a period of one to several nights around a full moon [137,141]. Polyps feed on a variety of small organisms, from microscopic zooplankton to small fish. These organisms are immobilized or killed by the poison carried in the nematocysts existing in the polyp’s tentacles, which is discharged in response to contact with another organism. The tentacles then manoeuvre the prey to the mouth and into the stomach [142].

#### 6.1.1. Coral-Derived Bone Grafts Substitutes

Coral-derived bone graft substitutes have attracted the interest of many experimental researches aiming at the characterization and selection of the most appropriate coral species for the intended applications, and at evaluating their in vitro and in vivo performances. The interest in this topic is also highlighted in a few review articles giving accounts of the literature reports published mostly in the last three decades [137,143,144]. The use of only natural or HA-derived corals, but also the combination of these porous structures with cells and/or growth factors, have been explored. The various coral bone graft substitutes currently available for experimental and biomedical applications and ongoing investigations of coral-derived bone replacement materials are summarised elsewhere [145,146]. *Porites*, *Goniopora*, and *Montipora* digitate, also known as finger coral, are some of the most common coral species exploited in medical applications [144,145,146]. *Porites* species possess an anatomical structure, and physical and chemical characteristics that more closely simulate the cortical bone, with a porosity <60%, and interconnecting pore sizes of ~190 μm, the average diameter of an osteon in human bone. The structure of *Goniopora* more closely resembles that of cancellous bone, with a porosity >70% and larger pore sizes [144,145].

##### Natural and Partial Transformed Corals

Sergeeva et al. [147] studied the cytocompatibility and biocompatibility of five coral scaffolds derived from *Acroporidae* and *Pocilloporidae*. Cytocompatibility was in vitro evaluated using human fibroblasts and by the formazan assay (MTT), and their biocompatibility was in vivo studied by implantation of the scaffolds into bone defects in rats. All of the specimens were cytocompatible and biocompatible. A comparison between a coral and autograft was accomplished by Puvanesway et al. [148], with the aim of studying their morphological and chemical composition, as well as the osteogenic differentiation potential in vitro using rabbit MSCs. The SEM analysis of bone and coral grafts revealed interconnected pores, and micro-CT measurements confirmed pore sizes in the range of 107–315 μm and 103–514 μm, respectively, with total porosity fractions >92%, which seems to be exaggerated in comparison to other reported values [146]. Significantly higher levels of osteogenic differentiation markers, namely, ALP and OC, and of ON and Runx2 integrin gene expression, were detected in the coral graft cultures in comparison with those in the bone graft cultures. The authors concluded that coral grafts enhanced the osteogenic differentiation of rabbit MSCs relative to the bone graft culture system.

Mangano et al. [149] used calcium carbonate in sinus elevation procedures and evaluated its clinical performance through histologic and histomorphometric analysis. After a six-month post-implantation period, the mean vertical bone gain was about 7 mm and the histomorphometric analysis revealed a residual calcium carbonate of ~15%, ~28% of newly formed bone, and ~57% of marrow spaces. The reported implant survival rate after one to five years of follow-up was 98.5%. The osteoconductivity and suitability of coral-derived calcium carbonate was compared with S53P4 bioactive glass and allogeneic fresh frozen bone by Gunn et al. [150] through the implantation of the material into cylindrical bone defects drilled in the femoral condyles of adult rabbits. Histologic and histomorphometric analyses were performed at 3, 6, 12, and 24 weeks. All three materials were found to be biocompatible and osteoconductive. Coral was observed to degrade more quickly, leaving more empty space in the defects, being considered the least suitable bone filler, with no statistically significant difference being observed between the allograft and the bioactive glass.

As previously mentioned, calcium carbonated coral skeletons can be hydrothermally converted into CaP scaffolds. In this regard and in order to understand whether coral-HA can be a promising alternative to intraarticular autologous structural bone grafts, Koëter et al. [151] filled a defect in the femoral trochlea of goats with a coral-HA scaffold. They showed that coral-HA did not cause an inflammatory reaction and there was good bone growth in the defect filled with the scaffolds.

The potential of coral-HA, along with other biomaterials such as cryopreserved bone allograft (CBA), demineralized freeze-dried dentin (DFDD), and cementum, for periodontal regeneration was studied by Devecioğlu et al. [152]. The authors studied in vitro the adhesion, proliferation, and mineralization of periodontal ligament (PDL) cells and mouse embryonic pre-osteoblasts cells (MC3T3-E1). Both the CBA and coral-HA exhibited a better initial PDL cell adhesion and regarding the long-term PDL cell adhesion, there was an increase in the presence of coral-HA. In the tests with MC3T3-E1 cells, the mineral-like nodule formation was significantly higher in DFDD biomaterial. According to the authors, the overall outcome was good biocompatibility with both types of cell for all the analysed biomaterials.

Recognizing that the incorporation of additional elements in the scaffolds could improve their properties, Zhang et al. [153] combined silver with coral-HA, aiming to introduce antibacterial properties to the scaffold. The scaffolds were prepared through an adsorption process at the surface and an ion-exchange reaction between the Ag^+^ from silver nitrate and Ca^2+^ from the coral-HA. It was observed that the scaffolds’ morphology is dependent on the Ag^+^ concentration. The scaffold cytocompatibility was analyzed using MC3T3-E1 cells and it was demonstrated that cell morphology and proliferation is dependent on the Ag^+^ concentration; for instance, better results were achieved with lower Ag^+^ concentrations [(13.6 µg/mL)/coral-HA and (1.7 µg/mL)/coral-HA]. Importantly, the scaffold that combined silver with coral-HA exhibited an excellent biocidal potential against both Gram-negative (*Escherichia coli*) and Gram-positive bacteria (*Staphylococcus aureus*).

As stated earlier, corals are potential scaffolds for bone tissue engineering; however, their excessive use may damage their natural habitats. In this regard, Mahanani et al. [154] mimicked coral structure and studied their capacity for MSC adhesion and proliferation. The synthetic scaffolds were prepared from bovine gelatin and calcite CaCO_3_ powder with a 10% *w*/*v* solid concentration. They observed that MSC exhibited a good adhesion ability and when Platelet Rich Plasma (PRP) was incorporated into the scaffolds, the cell proliferation improved.

##### Natural and Partial Transformed Corals Combined with Mesenchymal Stem Cells

Manassero et al. [155] studied the potential of Acropora coral scaffolds for MSC delivery in an animal model. Upon in vitro cell adhesion and proliferation, the coral scaffolds were placed into a critical bone defect in sheep. They observed an almost complete scaffold resorption six months after the operation which, consequently, is associated with bone regeneration. The authors concluded that the presence of MSCs is beneficial for osteoinductive behaviour. A comparation between Acropora or Porite coral granules combined with MSCs for their potential use in bone regeneration was performed by Decambron et al. [156]. The cells were seeded on both types of coral granules, placed in a perfusion bioreactor, and then implanted into bone defects in sheep. They observed that despite an early resorption of coral scaffolds that led to a bone non-union, a superior bone formation was registered for Acropora scaffolds. Further, the former scaffolds resorbed slowly when compared to Porite scaffolds and, thereby, are more closer to the clinical use. Moreover, the osteogenic potential of these two coral species (*Acropora* and *Porites*) was compared with β-TCP scaffolds and banked bone in the presence or absence of MSCs. Bone formation was only registered in the samples containing MSCs and the coral scaffolds demonstrated the best bone formation capability [157].

The osteogenic potential of human BMSCs to induce bone formation even in ectopic sites has been already demonstrated in BMSC-soaked coral or HA implants in intramuscular pockets in rats [158] and in the repair of critical-sized mandibular defects in large mammals [159,160]. Similar conclusions were drawn by the same research group when coral scaffolds seeded with BMSCs were utilized [161] instead of β-TCP scaffolds used in the previous study [159]. Defects treated with coral alone were used as an experimental control. The engineered bone with coral/BMSCs achieved satisfactory biomechanical properties at 32 weeks post-operation, which was very close to that of the contralateral edentulous mandible. This contrasted with minimal bone formation with an almost solely fibrous connection in the group treated with coral alone [161].

Considering that adipogenic and osteogenic cells share part of the early differentiation cascade of MSCs and when compared to BMSCs are easily isolated, in relative abundance, and rapidly expanded, adipocytes have been used for the repair and regeneration of different tissues. For instance, Ruth et al. [162] investigated whether adipocytes that have initiated differentiation along one lineage could be converted into an osteogenic lineage by merely interacting with marine corals (*Porites lutea*). Through morphological, histological, enzymatic, and quantitative PCR analyses made at different time points (1, 2, 5, 7, 14, 21, and 28 days post-seeding), they demonstrated that preadipocytes could differentiate into bone-forming cells when grown on a biomatrix of marine origin without the addition of other bone morphogenesis inducers. Following the same line, Cui et al. [163] loaded autologous adipose tissue-derived stem cells (ASCs) onto natural coral scaffolds and investigated their potential for a cranial bone defect in a canine model. After 12 weeks of ASC-coral implantation, it was already possible to observe new bone formation and upon 24 weeks, 84.19 ± 6.25% of the defect had been repaired, whilst in the control group, coral alone, only 25.04 ± 18.82% was repaired. In a more recent work, the same authors studied the hypothesis of healing the same defect with allogeneic ASCs seeded onto coral scaffolds without the need of immunosuppressive therapy. They observed that the use of allogenic osteo-differentiated ASCs did not cause any significant systemic immune response or local inflammation and, importantly, micro-computed tomography (micro-CT) analysis demonstrated that the newly formed bone was analogous to that of autologous osteo-differentiated ASCs [164].

The capacity to develop a vascularised coral scaffold seeded with marrow-derived osteoblasts was analysed by Chen et al. [165]. Prior to the implantation, the scaffolds were in vitro incubated for two days. To obtain a vascularised biomaterial, the scaffolds were implanted under the rabbit inferior epigastric blood vessels. After two months of operation, a well-vascularised scaffold was obtained, for which new bone formation was observed by histological analysis.

Bensaïd et al. [166] compared natural coral with coral-HA, and both were seeded with MSCs. Firstly, in the natural coral scaffolds, it was observed that the presence of a pseudo-periosteal layer of MSCs involving the scaffold improved bone formation in comparison with a distribution of MSCs over the implant. Nevertheless, due to the high resorption rate of natural coral scaffolds, successful bone formation was only observed in one sheep. On the other hand, coral-HA combined with the MSCs layer and the autologous bone graft demonstrated similar new bone formation upon four weeks of implantation. Furthermore, after 14 months of implantation, the defects filled with coral-HA combined with the MSCs layer were completely replaced by new bone.

Gao et al. [167] wrapped cylindrical coral scaffolds around partially mineralised and strong osteogenic cell sheets that could be obtained from BMSCs under specific cell culture conditions. These cell sheets/scaffolds were implanted into subcutaneous pockets on the backs of mice and bone formation was studied by CT scanning and histological observation. Cortical bone was formed within the cell sheet/scaffold. After eight and 12 weeks of implantation, the bone formation was 26% and 40%, respectively. Histological observation showed that neo-bone formation occurs in the manner of endochondral ossification. The authors concluded that a partially mineralised and osteogenic cell sheet could vitalise a coral scaffold for bone formation. Following a similar strategy, Geng et al. [168] combined MSCs sheets and coral particles. Coral particles were incorporated on the surface of confluent rabbit MSCs, forming a cell sheet. Posteriorly, a tubular bone graft was obtained by wrapping the cell sheet around a mandrel. The obtained bone graft was cultured in a spinner-flask bioreactor and then implanted into subcutaneous pockets on the backs of nude mice. The authors observed that following the in vitro incubation period, the bone graft still exhibited a tubular shape, sufficient radiological density, and suitable values of compressive strength. The in vivo results demonstrated that the cell sheets had a good osteogenic capacity and after eight weeks of implantation, a mature tissue with a similar mineral density as the one of the mouse spine. 

In a different approach, Chen et al. [169] inserted a titanium dental implant into a coral scaffold seeded with BMSCs and then subcutaneously implanted the set into nude mice backs. Defects treated with coral carrying no cells were used as an experimental control. After two months of implantation, the local area of implantation was red and similar to native bone and, in addition, the scaffold was mostly absorbed. Also, dental implants were fixed in the newly formed bone and surrounding the implant, new bone was formed. On the other hand, no bone formation was observed in the control scaffold.

It has been recognized that in supra-alveolar defects, periodontal and bone regeneration could be improved when using guided tissue regeneration (GTR) due to the space provision, besides their osteconductive properties [170]. Wikesjö et al. [171,172,173,174] carried out different works in which supra-alveolar periodontal defects were created in young adult beagle dogs with the aim of evaluating space provision, alveolar bone, and cementum regeneration using natural corals in combination with GTR. An histometric analysis with different parameters [defect height, defect area, membrane height, junctional epithelium, connective tissue repair, cementum regeneration, bone regeneration (height, area, and density), and biomaterial density] demonstrated that for all the evaluated parameters, no significant differences could be observed among three different step-serial sections. Thereby, representative histometric data could be obtained from a central section [172]. The combination of coral/GTR was compared with coral [173] or GTR alone [174]. At four weeks post-operation, the histopathologic and histometric analysis revealed significantly increased bone formation (height and area) at sites receiving the coral/GTR combination compared with coral or GTR alone [173,174].

##### Natural and Partial Transformed Corals Combined with Growth Factors

The capacity of bone tissue to repair and regenerate is in part related to the direct differentiation of MSCs into osteogenic cells. In this differentiation process, different hormones and differentiation factors play fundamental roles. The osteoclasts have the ability to activate latent TNF-β and, subsequently, during bone resorption, the active TNF-β is released, thereby promoting osteoblast and bone formation [175]. Based on this concept, Vuola et al. [176] studied the effect of adding TNF-β1 into natural coral scaffolds implanted in defects created in parietal bone of Wistar rats on bone formation, using coral scaffolds without added TNF-β1 as controls. Indeed, the presence of TNF-β1 was said to improve bone formation, but the new bone was located around the implanted material and did not fill the defect. Furthermore, the addition of the growth factor delayed bone resorption and the authors associated that with the absence of fibrous tissue ingrowth and the decrease of the number of macrophages and giant cells.

BMPs are proteins present in the bone matrix and are able to induce chondrogenesis and osteogenesis. These molecules are associated with the first signal for the beginning of MSC differentiation and enhance osteoblast differentiation and osteoblastic differentiation. IGFs are also present in the bone matrix and play a role in bone formation. IGFs stimulate chondrogenesis and osteogenesis, as well as the synthesis of bone collagen by bone cells [175]. In this regard, Nandi et al. [177] incorporated BMP-2 or IGF-1 into HA scaffolds derived from coralline and used a rabbit model to study their potential for bone regeneration. Through an in vitro study, they observed that IGF-1 exhibited a more sustained release when compared to BMP-2. After 28 days, the release of IGF-1 and BMP-2 was 77 and 98%, respectively. The in vivo results demonstrated that the addition of growth factors improved the early-stage bone formation, though IGF-1 was demonstrated to be more effective.

Vascular endothelial growth factor (VEGF) is the best-characterized growth factor for the regulation of vascular development and angiogenesis. Since angiogenesis influences osteogenesis, VEGF is important in bone formation and remodelling [178]. Recognizing that the vascularization and osteogenesis of block grafts still remains a key problem for dentists, Du et al. [179,180] attempted to promote angiogenesis and prevascularization by seeding nano-HA (nHA)/coral blocks with angiogenic recombinant human vascular endothelial growth factor_165_ (rhVEGF_165_). Non-coated and coated drafts were implanted in mandibular critical-size defects using male beagle dogs as an animal model. The histological evaluation and the histomorphometric analysis revealed enhanced neovascular density and a larger quantity of new bone formation at three and eight weeks post-surgery. The results suggest that nHA/coral blocks might be satisfactory scaffolds for block grafting in critical-size mandibular defects and that additional VEGF coating via physical adsorption can promote angiogenesis in the early stage of bone healing.

##### Natural and Partial Transformed Corals Combined with Mesenchymal Stem Cells and Growth Factors

A bone tissue engineering strategy that combines a calcium-based scaffold and MSCs is beneficial for bone growth; however, it may lack osteoinductive properties. This can be overcome by the addition of growth factors. For instance, Xiao et al. [181] used autologous BMSCs from Beagle dogs and transfected them with adenovirus containing human BMP-2. Posteriorly, the BMP-2 expressing BMSCs were seeded onto a coral scaffold. The BMP-2-transfected BMSCs/coral scaffolds were placed in defects created in the canine medial orbital wall. The authors observed that a combined delivery of BMSCs and BMP-2 on the defect had the highest bone regeneration when compared with the defects treated with BMSCs/coral and only coral scaffolds. In a similar procedure, Tang et al. [182] transfected autologous BMSCs from the left tibia of osteoporotic rats with human BMP-2 but, instead of seeding them onto a natural coral scaffold, they used coral-HA scaffolds. Newly formed bone was observed four weeks post-implantation, while mature bone was reported to form upon eight weeks of implantation. It is important to highlight that BMP-2 in suprahysiologic doses causes significant adverse side-effects. Decambron et al. [183] combined coral scaffolds with MSCs and/or low-doses of BMP-2 and observed that dual delivery improved bone formation and bone union when compared to a single addition of MSCs or BMP-2 to coral scaffolds. A comparison of coral scaffolds combined with MSCs and human BMP-2 and autologous bone grafts was performed by Hou et al. [184]. BMP-2 was firstly added to each coral, followed by the subsequent seeding of MSCs. The grafts were implanted in critical defects of rabbit crania. A similar bone formation was registered in the defects treated with coral/BMP-2/MSCs scaffolds (77.45 ± 0.52% in radiopacity) and with autografts (84.61 ± 0.56% in radiopacity). In addition, the osteogenesis rate in coral/BMP-2/MSCs was significantly higher compared to coral/BMP-2 scaffolds. These authors highlight an allegedly synergetic effect between MSCs and BMP-2 seeded in the coral scaffolds. More importantly, these combined scaffolds were reported to exhibit a similar behaviour to autologous grafts.

bFGF is produced by osteoblasts and stored in ECM in an active form and, thereby it regulates bone remodelling. In addition, bFGF promotes the proliferation of endothelial cells and neovascularization [185,186]. With this in mind, Zheng et al. [187] studied the feasibility of seeding mandibular condyle constructs with BMSCs onto porous coral scaffolds. The human BMSCs were transfected with bFGF gene-encoding plasmids and induced to differentiate into osteoblasts and chondroblasts. The osteogenic/chondrogenic differentiation, cell proliferation, collagen deposition, and tissue vascularization were evaluated. They observed that the transfected human BMSCs (h BMSCs) expressed bFGF and were highly proliferative. Moreover, subcutaneous transplantation of seeded coral/hydrogel hyaluran constructs into nude mice resulted in bone formation and collagen type I and type II deposition. Neovascularization was observed around newly formed bone tissue; bFGF expression was detected in implanted constructs seeded with bFGF expressing hBMSCs. Based on the encouraging results, the authors suggested that engineered porous coral constructs seeded with bFGF gene-transfected hBMSCs may be a feasible option for surgical transplantation in the temporomandibular joint.

Zhang et al. [188] prepared porous chitosan/coral composites combined with a plasmid encoding the PDGF-B gene through a freeze-drying process. PDGF-B is an important growth factor for wound healing, as well as for promoting the recruitment and proliferation of PDL and bone cells [189]. The scaffolds were evaluated in vitro by analysis of the microscopic structure and cytocompatibility. The expression of PDGF-B and ColI were detected after seeding human PDL cells in the chitosan/coral scaffold composites. The subcutaneous implantation of these scaffolds into mice revealed that the proliferation properties of hPLCs on the gene-activated scaffolds were much better than on the pure coral scaffolds. The expression of PDGF-B and ColI was also superior in gene-activated scaffolds. The results of this study suggest the porous chitosan/coral composite scaffolds combined with the PDGF-B gene as a potential construct for periodontal tissue regeneration.

Gross-Aviv et al. [190] studied the influence of the coral surface chemistry on the differentiation of MSCs. For this purpose, an aragonite matrix derived from the coral *Poris lutea* and a gold-coated *Poris lutea* were seeded with MSCs and combined or not combined with growth factors (TGF-β1 and IGF-I). An aragonite surface promoted osteogenic differentiation and, on the other hand, the gold coating that prevented the contact between the cells and the aragonite surface led to chondrogenic differentiation. The authors stated that the chondrogenic differentiation on the gold-coated scaffolds is associated with the inability of direct contact between the Ca^2+^ environment of the scaffold and the MSCs. Furthermore, the supplementation of the culture medium with the growth factors increased the influence of the surface chemistry on the cell differentiation.

Xiao et al. [191] studied the effects of osteogenic, BMP-2, and angiogenic, VEGF, factors on the repair of critical-sized bone defects in rabbit orbits. They used autologous BMSCs genetically modified to express human BMP-2 and VEGF165, which were then seeded on natural coral scaffolds. The defects were filled with coral scaffolds that had been loaded with non-transfected or transfected BMSCs with a single or combined growth factors. After four weeks of implantation in critical defects, those filled with the coral scaffolds with a combined delivery of BMP-2 and VEGF were reported to exhibit improved angiogenesis. Moreover, a maximum rate of bone formation was registered before the eighth week of implantation and a total bone-union was observed at the 16th week of implantation. The authors concluded that the presence of both BMP-2 and VEGF improved the angiogenesis and bone regeneration. Further, there was a synergetic effect between VEGF and BMP-2 during bone formation.

PRP extracted from autologous whole blood contains a wide range of autologous growth factors, namely PDGF, TGF-β, IGF-1, VEGF, and bFGF. It is beneficial for tissue healing and is being broadly used in oral and maxillofacial surgery [192,193]. With this in mind, Zhang et al. [194] studied the effect of clotting natural porous coral disks with PRP on the bone formation of marrow stromal cells (MSCs). The samples were cultured in vitro or implanted subcutaneously into nude mice. Coral scaffolds loaded with MSCs or with PRP alone were used as controls. The levels of ALP, a marker enzyme of bone formation activity, were measured for the specimens cultured in vitro for seven and 14 days, while the levels of ectopic bone formation were evaluated four and eight weeks after the operation. The in vitro results revealed that the samples from the coral/PRP/MSC group exhibited significantly higher ALP activity compared with those from the coral/MSC group or the coral/PRP group. The histomorphometric analyses of the in vivo experiments also showed higher levels of new bone and/or cartilage formation in the coral/PRP/MSC group, four and eight weeks after implantation, in comparison to the control specimens. The authors concluded that PRP could improve the ALP activity of MSCs on coral and increase ectopic bone formation.

### 6.2. Cuttlefish Bone

**Figure 5:**
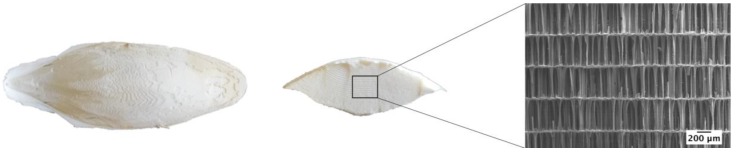
CB bone, its tranverse section and the scanning electron microscopy of the dorsal shield and the lamellar matrix.

The cuttlefish, Sepia officinalis, is a common demersal neritic species occurring predominantly near sandy and muddy bottoms up to a depth of 200 m. CB represents approximately 9% of the cuttlefish and is a hollow structure divided by lamellae (Figure 5) [22,195,196]. Besides functioning as a skeleton, the porous structure contains liquid and gas (mainly nitrogen at a pressure of about 0.8 atm) that provides neutral buoyancy to the cuttlefish by varying its density [195,196]. Through the liquid movement into or out of the CB via osmotic forces, the volume fraction of gas in the bone changes and the cuttlefish might become more or less dense than the sea water. This buoyancy mechanism, which is almost independent of depth, confers a valuable advantage to the cuttlefish since it can maintain a fixed position in water with little effort [195,197]. This buoyancy mechanism is more stable than that conferred by the swim bladder of a fish, which leads to a state of unstable equilibrium when adapting to the depth. Indeed, if the fish rises in the sea, the swim bladder will expand and tend to push it still higher, whereas if it goes deeper in the sea, the swim bladder will be compressed and the fish will tend to sink more and more quickly [197]. To accomplish these functional and highly sophisticated requirements, the high porosity of CB enables the cuttlefish to maintain its neutral buoyancy at a higher depth and at the same time retain enough stiffness and strength to prevent sever distortion or crushing under high hydrostatic pressures in deep water [195,198,199].

The dorsal shield and lamellar matrix represent the two main components of CB. The dorsal shield is highly dense and provides a rigid substrate for protecting the development of the lamellar matrix. On the other hand, the lamellar matrix has a high porosity, approximately 93%, and also manages to withstand very high hydrostatic pressure [199].

The dorsal shield is a dense and tough cover that overlays the lamellar matrix and seals off the separated chambers. It consists of a non-porous structure containing 30 to 40% by weight of organic material, β-chitin. This organic component has the ability to undergo plastic deformation, thereby increasing the toughness of the structure. It is composed of three different layers: a 1 mm thick outer calcified layer; a 0.3 mm thick transparent middle zone composed of tough, fibrous layers of sclerotized chitin; and a thin inner calcified zone [195].

The lamellar matrix is a layered, quasi-periodic microstructure composed of lamellae separated by pillars [200], which is able to resist external pressures of about 2.4 MPa [195]. The spacing of the lamellae varies between 200 and 600 µm along the CB [196], being dependent on the species [201]. Each chamber is supported by pillars that form channels with a high degree of interconnectivity, and a uniform width that progresses through the material along a sigmoidal path [199]. Its unique macroporous architecture with approximately 93% porosity is mainly composed of aragonite, which is a crystallized form of CaCO_3_. This inorganic structure is enveloped in a thin layer of organic material primarily composed of β-chitin that represents between 3% and 4.5% of the lamellar matrix weight [195,199,202]. The presence of β-chitin is associated with initiation, organization, and inhibition of the inorganic matrix mineralization [195,202]. Moreover, the organic matrix may function as a template for the nucleation of the inorganic material, possibly due to the interaction between the groups of the polymer and the ions in the solution phase or the nuclei of the solid phase that become more stable upon bonding [195].

CB has a unique architecture which is associated with a high ratio of compressive stiffness to weight. Indeed, under excessive compressive loading, CB can collapse in a controlled and layer-by-layer manner due to its layered structure and also to the sigmoidal shape of its pillars. In this regard, the cuttlefish can stay below the depth limit of its CB for short periods of time without significant damage. The plastic deformation of the organic component in the dorsal shield provides another important contribution to the toughness of the structure [195,199].

#### 6.2.1. Cuttlefish Bone-Derived Bone Grafts Substitutes

CB is a worldwide available and inexpensive material with a unique porous microstructure; the pore size and pore interconnectivity of which is proven to be beneficial for bone growth and vascularization [203]. The use of CB as a bone graft substitute has been reported not only in its natural form, but also as an aragonite source, to prepare CaP materials by preserving the same porous structure after hydrothermal transformation.

##### Natural Cuttlefish Bone

Dogan et al. [204] studied the potential of CB as a xenograft by filling a bone defect of a rabbit with CB, demineralized bone matrix (DBM), bovine cancellous graft (BCG), and TCP. At the third post-operative week, 100% callus formation in the groups treated with CB, DBM, and TCP was observed. In the animal treated with CB, remodelling could be observed at week 6, while in the other groups, remodelling was only observed upon the twelfth week. In addition, in the group treated with CB, fibrous union started at the first post-operative week and the rate of vascularization was higher when compared to the other groups. The osteochondral union at week three resulted in an increase of the development of new bone. The defects treated with CB or with TCP exhibited the fastest remodelling. Overall, in the CB group, a high degree of vascularization and good osteogenesis and osteointegration properties were observed. Thus, CB was considered a good xenograft material.

As previously mentioned, CB is composed of a dorsal shield and lamellar matrix. Kim et al. [205] separated the CB into these two structures and studied whether the presence of small amounts of heavy metals on CB could influence its biocompatibility and osteoblast differentiation of human MSCs. It was observed that the heavy metals did not affect the cell viability and CB allowed cell adhesion and growth. When compared to the dorsal shield, in the lamellar matrix, the cells infiltrated deeper into the structure. ALP levels increased on both the dorsal shield and lamellar matrix; however, a higher expression was observed on the dorsal shield due to the fact that confluence is achieved earlier in this material and this, consequently, resulted in earlier differentiation. Collagen type I α1 (ColIα1) and Runx2 were increased on both materials with no significant difference.

Besides being used as a scaffold, natural CB can also be milled and used to fill poly (methyl methacrylate-*co*-styrene) bone cements [206,207]. When compared with the non-filled cement, the addition of 10 and 30 wt.% of CB resulted in an increase of tensile strength and Young’s modulus. Nonetheless, a cement containing 50 wt.% of CB led to a decrease in tensile strength and Young’s modulus, which was associated with the formation of pores. Although the compressive strength decreased with the increase of CB content, it maintained a higher value than the minimum requirement of 70 MPa [206]. Due to the exothermic polymerization reaction, there is an increase of temperature as the reaction progresses, decreasing after a maxim value has been reached as the monomer is consumed. Due to its thermal capacity, the increase of CB content led to a decrease of the peak temperature and an increase of the time to reach that temperature, resulting in an apparent slower reaction. Furthermore, the setting time increased with the CB content, which is beneficial as it allowed enough manipulation time before setting. However, a longer setting time can cause some medical problems because pressure in the prosthesis has to be maintained until the cement sets [207]. When implanted in bone defects, the cements without CB were weakly adherent to the parietal bone, while the samples containing CB were strongly attached to the bone, an indication that osteointegration has occurred in samples containing CB [206].

##### Calcium Phosphate Materials Derived from Cuttlefish Bone

Being mainly composed of aragonite and having a suitable porous microstructure for bone grafting, which is preserved upon hydrothermal transformation into CaP, CB has been used as an interesting source of calcium carbonate and template for preparing CaP scaffolds or powders.

The transformation of CB into HA through a hydrothermal process was first reported by Rocha et al. [138,203,208]. Prior to the transformation, the exact aragonite content in CB was analysed and the corresponding amount of (NH_4_)_2_H_2_PO_4_ was added to set the molar ratio of Ca/P = 1.67. The mixture was sealed in a polytetrafluoroethylene (Teflon) lined stainless steel autoclave and the hydrothermal treatment (HT) was conducted at 200 °C. The CB internal structure was not compromised during the HT and thereby the pore size and interconnectivity were maintained after the transformation. An AB-type carbonated HA was obtained, which is similar to the composition of human bones [136,208]. Additionally, the size of HA crystallites was similar to the size of bone-like apatite (20–50 nm) [136]. The obtained scaffolds were demonstrated to have a high thermal stability on sintering up to 1350 °C. Above 1400 °C, it was possible to observe the formation of β-TCP. Moreover, scaffolds were demonstrated to have a very good in vitro bioactivity due to the fact that when immersed in SBF, there was a rapid and evident formation of an HA layer [203]. In the presence of the scaffolds, the viability and proliferation increased, and the ALP activity was maintained at a constant value, indicating that the scaffolds were biocompatible with osteoblasts [203,208].

Different HT conditions were established by Hongmin et al. [209], who used a 0.5 M (NH_4_)_2_HPO_4_ solution and a CB/(NH_4_)_2_HPO_4_ weight ratio of 1/1.2, and the treatment was conducted at 180 °C for 96 h. The authors compared the in vitro and in vivo behaviour of HA scaffolds derived from CB (CBHA) and raw CB. The protein adsorption was higher on CBHA than on CB. Despite the good cell adhesion and proliferation on both CBHA and CB, CBHA demonstrated a better capacity for cell differentiation. Indeed, after 13 days of culture, ALP activity and the OC level were significantly higher on CBHA. Regarding the in vivo behaviour, both scaffolds were encapsulated in a fibrous tissue at the fourth week; however, a higher quantity of fibrous tissue and blood vessels was observed on CBHA. At week eight and contrarily, osteoblasts were found to excrete bone matrix and embed themselves to form bone lacunae. In this way, it was possible to conclude that CBHA represents an interesting osteoinductive bone graft.

As previously observed, different HT approaches can be followed to obtain CaP materials from CB. In this regard, Ivankovic et al. [200] and Kasioptas et al. [210] studied the mechanism and kinetics of the aragonite transformation into CaP scaffolds by modifying the temperature and time of the hydrothermal reaction. Ivankovic et al. [200] analysed a range of transformation temperatures from 140 to 220 °C for various time periods between 1 and 48 h. In the samples treated at 140 and 160 °C for 20 min, they observed the presence of CaHPO_4_·2H_2_O (brushite), a compound that preferentially precipitates at lower values of pH and temperature. A complete transformation was only observed upon the HT at 200 °C for 24 h. At 180 °C, the transformation is not completed, even after 48 h of HT. On the other hand, the formation of CaHPO_4_ (monetite) was reported to occur at 220 °C and above 4 h of HT. The isothermal kinetics of transformation was described by the Johnson-Mehl-Avrami (JMA) equation (Equation (1)), which is often used to describe the nucleation and growth of crystals from amorphous materials.

(1) $$\alpha =1-exp\left[-(k^{n}\;{\left(t-\tau \right)}^{n})\right]$$

In Equation (1), α represents the fraction of transformed HA; *k* is the rate constant; *n* is the Avrami exponent, associated with the nucleation type and growth dimensions; and *τ* is the incubation time. The rate constant increased with the increase of temperature; however, the Avrami exponent was maintained at an almost constant value and around 0.5 over the entire range of temperatures tested, indicating that the growth mechanism of HA can be defined as one-dimensional growth controlled by a diffusion process.

Alternative methods for HT can be used to produce CaP materials derived from CB. For instance, Sarin et al. [211] reported the production of BCP scaffolds from CB using a solution of phosphoric acid and 2-propanol with different concentrations of phosphoric acid from 12 to 20 wt.%. Briefly, the infiltration of the solution into the CB occurred under vacuum conditions and for 1 h. This process was followed by heat treatment at high temperatures, up to 1300 °C. The BCP scaffolds obtained with 16 wt.% of phosphoric acid preserved the initial microstructure of CB and exhibited a compressive strength of 2.38 ± 0.24 MPa that, despite being lower than the raw CB (2.74 ± 0.39 MPa), is in the range of trabecular bone. Dutta et al. [212] produced the CaP scaffolds derived from CB in ambient conditions using a de-calcification, re-calcification process followed by phosphate mineralization. CaCO_3_ was removed from the CB structure by dipping the CB in acetic acid. The as-obtained organic matrix was re-mineralized using solutions of calcium chloride and sodium carbonate and hence amorphous calcium carbonate (ACC) was produced, due to the fact that macromolecules associated with biominerals have the capacity to stabilize ACC. Afterwards, ACC was converted into CaP by immersing the mineralized scaffold into a phosphate solution followed by a treatment with glutaraldehyde solution. Since the solubility of ACC and calcite at the working pH of 7 is high, there was a complete phase conversion, not to HA, but instead to brushite, the formation of which is favoured at this pH.

In addition to being converted into porous CaP scaffolds preserving the internal structure, CB can also be used to produce CaP powder [213,214] or granules [215]. Lee et al. [213] mixed CB that was previously calcined, consisting of pure CaO phase, with phosphoric acid, to synthesize CaP. Different ratios between CB and phosphoric acid from 1:1.0 to 1:1.7 were tested. HA and β-TCP were obtained at 1:1.2 and 1:1.7 ratios, respectively. The samples heat treated at 900 °C were entirely crystalized. CBHA granules with a size varying from 200–500 µm were obtained by HT at 200 °C for 24 h. Besides being non-toxic, the CBHA granules enabled a higher cell density and improved cell differentiation capacity in comparison to pure HA granules. This could be justified not only by the higher surface roughness and surface area of CBHA that are beneficial for cell proliferation and adhesion, but also because CBHA contains some mineral ions, like magnesium, which play fundamental roles in the binding of cell-surface receptors and ligand proteins that, consequently, can enhance cell attachment. The in vivo results were in agreement with the in vitro tests and CBHA had a significantly higher percentage of bone formation and more multi-nucleated giant cells, fibroblasts, osteoblast-like cells, and connective tissue and micro blood vessels [215]. Furthermore, Kim et al. [214] produced a porous composite scaffold by solvent casting and particulate leaching using CBHA powder and PCL. CBHA powder, obtained by HT at 200 °C for 24 h, and PCL were mixed with salt particles (200–300 µm) that acted as a porogen. The introduction of CBHA into the PCL scaffolds improved the compressive strength. Regarding the in vitro tests, the addition of CBHA improved the viability, adhesion, and proliferation. In the in vivo studies, it was demonstrated that CBHA/PCL scaffolds improved the bone formation.

##### Doped—Calcium Phosphate Materials Derived from Cuttlefish Bone

The inorganic part of the human bone consists of carbonated HA with trace amounts of other ions like Na^+^, Mg^2+^, Zn^2+^, Sr^2+^, F^−^, and silicon. Therefore, the incorporation of the aforementioned ions into the CaP matrix has been used to improve the biological performance of the material. With this in mind, Kannan et al. [216] and Kim et al. [217] incorporated F^-^ and silicon (Si) ions into the CBHA structure, respectively.

F^−^ is an important element for bone and dental growth. It is involved in the prevention and treatment of dental caries. The incorporation of F^−^ ions into the HA structure stimulates the osteoblast proliferation and differentiation and improves the mineral deposition in cancellous bone [218,219,220]. Two different levels of F^−^ substitutions (46% and 85%) on the OH^−^ sites via HT at 200 °C for 24 h were studied by Kannan et al. [216]. For the HT, a required volume of (NH_4_)_2_HPO_4_ was added to the CB samples setting a Ca/P molar ratio of 10/6 and F^−^ was added by the NH_4_F solution at 1 M or 2 M. A nano-sized AB-type carbonated apatite with precise control of F^−^ content was obtained. For a substitution of 85%, the lattice parameters were typical for fluorapatite, leading to a contraction along the a-axis.

Silicon represents an important trace element in bone, as it is associated with bone growth. The incorporation of Si in HA materials modifies their surface by creating a more electronegative surface, generating a finer structure, and also increasing the solubility. Furthermore, Si-substituted materials have been demonstrated to improve the bioactive behaviour and increase the bone formation in vivo [96,220]. Kim et al. [217] synthesized Si-substituted CBHA using hydrothermal and solvothermal methods. Firstly, CB and (NH_4_)_2_HPO_4_ were placed in an autoclave for HT at 200 °C for 6 h. Subsequently, CB was immersed in a solution of silicon acetate (Si(CH_3_COO)_4_) saturated with acetone and solvothermal treatment took place at 200 °C for 12 h. In the final step, CB was again mixed with a (NH_4_)_2_HPO_4_ solution and the HT occurred at 200 °C for 12 h. The Si content of the samples was about 0.77 wt.%. Regarding the in vitro results, the presence of an Si-OH layer on the biomaterial surface was associated with the improvement of cell adhesion and proliferation on the Si-substituted CBHA. Moreover, the incorporation of Si enhanced cell density and consequently improved cell differentiation, which was observed by the ALP activity and expression of Runx2, ColIα1, and OC. According to the in vitro results, the in vivo tests demonstrated that bone formation was higher on Si-substituted CBHA than on CBHA.

##### Composite Materials Derived from Cuttlefish Bone

CaP scaffolds obtained from CB are normally brittle and exhibit low strength, thereby limiting the application of these materials as bone grafts. In order to improve the compressive strength, some authors investigated the coating of these scaffolds with polymers like PCL [221,222,223,224], polyvinyl alcohol [222], and collagen [225].

CBHA scaffolds were obtained through an HT and PCL was incorporated into the scaffold structure under vacuum to remove the air from the pores and allow a complete impregnation of the polymer into the structure [221,222,223,224]. Kim et al. [221] reported the coating with 1, 5, and 10% of PCL of CBHA scaffolds. The PCL layer obtained by the coating with 1% PCL could hardly be observed and the roughness of the sample was similar to the uncoated one. Coating with 5% PCL led to the formation of a thin PCL layer that covered the entire surface. When further increasing the amount of PCL to 10%, a thicker layer was formed and the number of clogged pores increased, thereby decreasing the porosity. Nonetheless, Milovac et al. [223] coated the scaffolds with 20% PCL, and although roughness and porosity noticeably decreased, they reported that such a coating allowed the maintenance of the number of pores and their interconnectivity. Through an SBF assay, it was possible to observe the formation of an apatite layer on the PCL-coated scaffold [222]. The compressive strength decreased with the HT due to a more extensive degradation of the organic matter that provides the mechanical support of the structure; however, with the exception of the coating with 1% PCL, the compressive strength improved with the PCL coating, as reported in Table 2. The coated scaffolds did not exhibit any cytotoxicity and cells were able to adhere and proliferate, and it was also demonstrated that penetration of the cells happened through the entire depth of the scaffold [221,224]. The cell proliferation was higher for the sample coated with 5 and 10% PCL. However, for the sample coated with 10% PCL, the cell proliferation decreased after six days of culture due to the reduction of porosity and the consequent lower surface area available for cell adhesion [222]. The ALP activity increased on the scaffolds coated with 5, 10, and 20% PCL [221,224]. The expression of ALP, Runx2, and ColIα1 significantly improved with the coating of 10% PCL, since the cells reached the confluence earlier, which consequently beneficiated the differentiation of cells [221]. The scaffolds coated with 20% PCl exhibited a higher level of collagen production, suggesting that the PCL coating improved cell adhesion and consequently induced higher levels of collagen secretion [223].

Siddiqi et al. [222] reported the coating with 5% of PVA of CBHA. The addition of 5% PVA improved the mechanical strength to 0.95 MPa compared to the raw CB (0.61 MPa) and the hydrothermally-transformed CBHA scaffold (0.38 MPa). The coating with PVA did not modify the viability and the adhesion and proliferation capacity of CBHA scaffolds.

Alternatively to the production of CBHA through HT and a posterior coating with polymer, Sukul et al. [225] modified the raw CB with HA and collagen (CB-HA-COL). To this end, CB was immersed in SBF solution to form an HA layer and samples were subsequently coated with collagen through the freeze-drying method. The modification of the CB surface with HA and collagen resulted in lower values of pore size (80–100 µm) and porosity (~84%), but still, these values have been reported as beneficial in bone tissue engineering. On the other hand, the coating enhanced the compressive strength from 2.00 ± 0.40 MPa for raw CB to 2.71 ± 0.16 MPa for CB-HA-COL. The coated scaffolds promoted cell adhesion and proliferation and showed higher levels of ALP expression. 

## 7. Conclusions

The earlier industrial revolution, in addition to the innovations in agricultural production and distribution, enabled nutritional diversity and consistency to large numbers of people, increasing the survival rate. The progresses in medicine, namely related to the understanding and control of infectious and parasitic diseases, some of the common causes of death, and improvements in clinical practice, in addition to the noticeable changes in public health that occurred from the late 19th century and physical activity, have led to a gradual and remarkable increase in human life expectancy at birth. Not surprisingly, postponing death to old ages came together with several problematic health consequences related to aging, including degenerative diseases such as cancers and diseases of the circulatory system, as well as the brittleness and breakage of bones weakened by osteoporosis, especially for older women.

The increased incidence of degenerative diseases such as osteoarthritis, especially in cases of excessive sedentary behaviour, and bone tumour resections demands for suitable bone grafts. Although the autologous bone graft is the gold standard clinical material for bone regeneration because of its advantages in terms of osteoconduction and osteoinduction, the limited availability and donor site morbidity are the most obvious drawbacks related to this option. A bone allograft is an alternative source, being the second most used option for orthopaedic procedures due to the availability in various forms and large quantities. But the main limitations include reduced osteoinductivity that may lead to inferior healing as compared with the use of autologous grafts, and to potential risks of the immune response. Xenografts are abundant, but the potential rejection by the host remains and cannot be discarded.

The shortcomings of the biologically-derived bone grafts and the increasing demand for bone repair materials constitute the main driving forces for the continuous search of synthetic bone graft substitutes. This article reviews the state of the art of synthetic bone graft materials fabricated from synthetic calcium phosphate powders or resulting from the total or partial hydrothermal transformation of aragonite marine-derived porous structures such as corals and cuttlefish bones. Tailor-made porous structures can also be made by using additive manufacturing techniques. Doping the calcium phosphate materials with selected doses of other elements enhances the biological performance of the inorganic materials. On the other hand, combining the inorganic components with suitable biopolymers is a very promising strategy for obtaining multifunctional bone graft materials. Furthermore, applying tissue engineering principles by suitably combining growth factors and cells is a method employed for developing synthetic bone grafts with in vivo performances that compete with autografts.

## Figures and Tables

**Table 1:** Main calcium orthophosphate compounds [27,92,93,94,95].

Compound	Formula	*Ca/P* Molar Ratio	Mineral	Symbol
Monocalcium phosphate anhydrous	Ca(H_2_PO_4_)_2_	0.50	—	MCPA
Monocalcium phosphate monohydrate	Ca(H_2_PO_4_)_2_·2H_2_O	0.50	—	MCPM
Dicalcium phosphate anhydrous	CaHPO_4_	1.00	Monetite	DCPA
Dicalcium. phosphate dihydrate	CaHPO_4_·2H_2_O	1.00	Brushite	DCPD
Octacalcium phosphate	Ca_8_H_2_(PO_4_)_6_·5H_2_O	1.33	—	OCP
Amorphous calcium phosphate ^1^	Ca_X_H_Y_(PO_4_)_Z_·nH_2_O	0.67–1.50	—	ACP
α-Tricalcium phosphate	α-Ca_3_(PO_4_)_2_	1.50	—	α-TCP
β-Tricalcium phosphate	β-Ca_3_(PO_4_)_2_	1.50	—	β-TCP
Calcium-deficient hydroxyapatite ^2^	Ca_10−x_(HPO_4_)_x_(PO_4_)_6−x_(OH)_2−x_	1.5–1.67	—	CDHA
Sintered hydroxyapatite	Ca_10_(PO_4_)_6_(OH)_2_	1.67	Hydroxyapatite	HA
Tetracalcium phosphate	Ca_4_(PO_4_)_2_O	2.00	Hilgenstockite	TTCP

^1^ The ACP normally has *x* = 3, *y* = 3, *z* = 2, and n = 3–4.5; 15–20% H_2_O. ^2^ *x* may vary between 0 and 1. When *x* = 1 (the boundary condition with Ca/P = 1.5), CDHA has the following composition: Ca_9_(HPO_4_)(PO_4_)_5_(OH).

**Table 2:** Compressive strength of raw CB, CBHA, and the different percentages of PCL coating on CBHA.

Ref	Compressive Strength (MPa)					
	Raw CB	CBHA	CBHA-1%PCL	CBHA-5%PCL	CBHA-10%PCL	CBHA-20%PCL
[221]	1.63 ± 0.13	1.11 ± 0.26	1.25 ± 0.56	2.32 ± 0.44	3.67 ± 0.46	—
[222]	0.609	0.376	—	1.376	—	—
[223]	0.46 ± 0.06	0.15 ± 0.09	—	—	—	0.88 ± 0.11
